# Evolution of the Transmission-Blocking Vaccine Candidates Pvs28 and Pvs25 in *Plasmodium vivax*: Geographic Differentiation and Evidence of Positive Selection

**DOI:** 10.1371/journal.pntd.0004786

**Published:** 2016-06-27

**Authors:** Ricardo A. Chaurio, M. Andreína Pacheco, Omar E. Cornejo, Ester Durrego, Craig E. Stanley, Andreína I. Castillo, Sócrates Herrera, Ananias A. Escalante

**Affiliations:** 1 Instituto Venezolano de Investigaciones Científicas, Caracas, Venezuela; 2 Institute for Genomics and Evolutionary Medicine (iGEM), Temple University, Philadelphia, Pennsylvania, United States of America; 3 Department of Biology, Temple University, Philadelphia, Pennsylvania, United States of America; 4 School of Biological Sciences, Washington State University, Pullman, Washington, United States of America; 5 School of Life Sciences, Arizona State University, Tempe, Arizona, United States of America; 6 Centro de Investigación Científica Caucaseco, Cali, Colombia; Johns Hopkins Bloomberg School of Public Health, UNITED STATES

## Abstract

Transmission-blocking (TB) vaccines are considered an important tool for malaria control and elimination. Among all the antigens characterized as TB vaccines against *Plasmodium vivax*, the ookinete surface proteins Pvs28 and Pvs25 are leading candidates. These proteins likely originated by a gene duplication event that took place before the radiation of the known *Plasmodium* species to primates. We report an evolutionary genetic analysis of a worldwide sample of *pvs28* and *pvs25* alleles. Our results show that both genes display low levels of genetic polymorphism when compared to the merozoite surface antigens AMA-1 and MSP-1; however, both ookinete antigens can be as polymorphic as other merozoite antigens such as MSP-8 and MSP-10. We found that parasite populations in Asia and the Americas are geographically differentiated with comparable levels of genetic diversity and specific amino acid replacements found only in the Americas. Furthermore, the observed variation was mainly accumulated in the EGF2- and EGF3-like domains for *P*. *vivax* in both proteins. This pattern was shared by other closely related non-human primate parasites such as *Plasmodium cynomolgi*, suggesting that it could be functionally important. In addition, examination with a suite of evolutionary genetic analyses indicated that the observed patterns are consistent with positive natural selection acting on *Pvs28* and *Pvs25* polymorphisms. The geographic pattern of genetic differentiation and the evidence for positive selection strongly suggest that the functional consequences of the observed polymorphism should be evaluated during development of TBVs that include Pvs25 and Pvs28.

## Author Summary

*Plasmodium vivax* is the most prevalent human malarial parasite outside Africa. The fact that patients can relapse due to the parasite dormant liver stages, among other biologic and epidemiologic characteristics of *vivax* malaria, facilitates the persistence of the disease in many endemic areas. These challenges have fueled the search for new control tools, including transmission blocking (TB) vaccines targeting the parasite sexual stages. Here we study the genetic diversity of two major TB vaccine antigens, Pvs25 and Pvs28. We show that these genes are relatively conserved worldwide but still harbor diversity that is not evenly distributed across the genes. These patterns are shared by the same proteins in closely related parasite species suggesting their functional importance. We also identify strong geographic differentiation between the circulating variants found in Asia and the Americas. Finally, evolutionary genetic analyses indicate that the observed variation in both genes could be maintained by natural selection. Thus, these polymorphisms may confer an adaptive advantage to the parasite. These results indicate that the genetic variation found in these genes and their geographic distribution should be considered by vaccine developers.

## Introduction

Transmission-blocking (TB) vaccines are considered an important tool for malaria control and elimination [1]. TB vaccines aim to disrupt malaria transmission by eliciting antibody mediated responses against antigens expressed during sexual or sporogonic stages of the parasite thereby inhibiting its development inside *Anopheles* mosquitoes. Thus far, the search of suitable targets for TB vaccines has yielded promising results. In particular, antibodies against some of the multiple parasite proteins have shown excellent TB activities [1]. Among those antigens, the ookinete surface proteins Pvs28 and Pvs25 have been considered candidates to be incorporated in TB vaccines against *Plasmodium vivax*. These proteins may have originated as result of a duplication event and their orthologous genes (referred to as *p28* and *p25*) have been described in many *Plasmodium* species [2,3]. P28 and P25 are the two most abundant membrane proteins expressed on the zygote and ookinete surfaces; indeed, they might represent as much as 25% of the total ookinete surface proteins [4]. Their structure has been characterized as a triangular prism of EGF-like domains tethered on the cell by a glycosylphosphatidylinositol (GPI) anchor at the C-terminus [5,6]. Although their specific functions are still not clear, it is known that they are essential for the survival of ookinetes in the mosquito midgut [4]. In particular, studies in *P*. *berghei* strongly suggest that P28/P25 proteins have multiple, and partially redundant functions during ookinete and oocyst development [4].

Although they share a common origin and their functions appear to overlap, preliminary studies indicate that P25 proteins are expressed earlier than P28. Specifically, P25 is expressed prior to fertilization, achieving peak synthesis in the initial hours soon after, and then most abundantly expressed on the surface of the developing zygotes and ookinetes [7]. In contrast, P28 proteins are expressed slightly later on the ookinete surface until the young oocyst stage [7]. In the context of developing TB vaccines, antibodies against these proteins interfere both with ookinete maturation and oocyst formation [8]. In particular, mice antisera against recombinant Pvs28 and Pvs25 recognized both antigens in short term cultures of parasite sexual-stages derived from patients with *P*. *vivax* malaria, and significantly suppressed oocyst development in four *Anopheles* species fed with blood infected with *P*. *vivax* Salvador I strain [8]. In addition, in a preclinical trial conducted in *Aotus* monkeys, animal immunization with recombinant Pvs25 elicited specific antibodies able to fully block parasite infection in membrane feeding assays (MFAs) [9]. Furthermore, in a phase I clinical trial conducted with Pvs25 sera obtained from the vaccinated volunteers induced significant inhibition of *P*. *vivax* transmission in *Anopheles dirus* mosquitoes using an *ex-vivo* MFA [1,10]. Moreover, TB immunity elicited with orthologous proteins in *P*. *falciparum* and other malaria parasites has been shown as well [8,11,12]. Unlike the extensive polymorphism commonly observed in several *Plasmodium* blood stage surface antigens, these proteins are considered to be conserved [13–17]. Therefore, the immunogenicity, TB potential, and limited polymorphism support the use of Pvs28 and Pvs25 as suitable targets for TB vaccines.

Here, we study a worldwide sample of *pvs28* and *pvs25* coding alleles. We detected strong geographic differentiation between populations in Asia and the Americas with replacements at specific amino acid residues novel in the Americas. We also found that these genes can be as polymorphic as some merozoite antigens such as MSP-8 and MSP-10, with most of their variation accumulating in the EGF2 and EGF3 like domains of both proteins. Finally, our analysis indicates that positive selection may be acting on the accumulation of *pvs28* and *pvs25* polymorphisms.

## Methods

### Parasite strains and field isolates

We report *pvs28* and *pvs25* complete CDS sequences from geographically and temporally diverse laboratory strains provided by William Collins at the Centers for Disease Control and Prevention (CDC). We obtained *pvs28* gene sequences from the following laboratory strains grouped by their geographic origin: Africa (Mauritania I), Central America (El Salvador II, Honduras III, Nicaragua, and Panama), South America (Río Meta from Colombia), Asia (Vietnam II, India VII, Thailand and Malaysia), and Oceania (Sumatra from Indonesia, Indonesia XIX, Chesson, and Harris from Papua New Guinea). We also obtained 11 *pvs28* and 15 *pvs25* sequences from Venezuelan archived samples [18]. In addition, we included 259 *pvs28* (total of 284 in the global alignment) and 310 *pvs25* (total of 325 in the final alignment) sequences available at the GenBank (release 208, June 2015). Those included data of *pvs25* from Venezuelan and laboratory strains [19] and sequences from *pvs28* and *pvs25* from China (Yunnan Province) [13], India (Delhi, Chennai, Kamrup, Nadiad and Panna) [16], Iran [17], Korea (ROK) [15], Southern Mexico [14], Thailand (Tak Province) [12] and, Bangladesh [20].

Additionally, we report 58 sequences for *p28* and *p25* orthologous genes from the following species of nonhuman primate parasites (NHPPs): *Plasmodium cynomolgi* (*p28* from: Berok, B, BX-20, Cambodian, *ceylonensis*, PT1, PT2, RO, Smithsonian, Gombak, and Mulligan strains; *p25* from: Berok, B, BX-20, Cambodian, *ceylonensis*, PT1, PT2, RO, and Smithsonian strains), *Plasmodium inui* (*p28* from: Celebes I and II, Leaf Monkey II, N34, OS, Philippine, Leucosphyrus, Perak, Taiwan I and II, and Perlis; *p25* from Celebes II, Hawking, Leaf Monkey I, Leucosphyrus, Mulligan, and Perlis), *Plasmodium knowlesi* (*p28* from: H, Hackeri, Malayan from Malaysia, Nuri from India, and the Philippine strain; *p25* from: Philippine, Hackeri, and Malayan), *Plasmodium coatneyi*, *Plasmodium fieldi* (*p28*: Hackeri and N-3; *p25*: ABI and N-3 from Malaysia), *Plasmodium hylobati*, *Plasmodium simiovale* (Sri Lanka), and a parasite from African primates, *Plasmodium gonderi*. Information about these species and strains can be found in Coatney *et al*. [21].

In order to estimate the phylogenetic relationships for the genes encoding *pvs28* and *pvs25* and their NHPPs orthologs, we also included the published sequences in PlasmoDB, version 24 [22] and NCBI for the Asian species *P*. *cynomolgi* (PCYB_062530, PCYB_062520) and *P*. *inui* (San Antonio 1, GCA_000524495.1 and; AY639974); the *Laverania* group that includes *P*. *falciparum* (3D7_1030900 and 3D7_10310003), *Plasmodium gaboni* (Pgk strain, GCA_000576715.1), and *Plasmodium reichenowi* (PRCDC_1030200 and PRCDC_1030300); the human parasite *Plasmodium ovale* (AB051632 and AB051631); and the rodent parasites *Plasmodium bergei* (strain Anka, AF232051 and XM_670232), *Plasmodium chabaudi* (AF232048 and XM_739934), and *Plasmodium yoelii* (AF232055 and XM_720005). The phylogenies were rooted with the avian parasite *Plasmodium gallinaceum* (M96886 and J04008). In the specific case of *P*. *cynomolgi* (strain B), in addition to the *p28* orthologous PCYB-062530, the two other paralogous genes were also retrieved (PCYB-062510/PCYB-007100) from PlasmoDB. To the best of our knowledge, only *P*. *ovale* [23] and *P*. *cynomolgi* [24] have one and two paralogs to *p28* respectively. In the specific case of *P*. *ovale*, we only included the *pos28-1* (AB051632) sequence in our phylogenetic analyses.

### PCR amplification, cloning, and sequencing

For all the samples processed in this study, DNA was extracted from whole blood by using QIAamp DNA Blood Mini Kit (Qiagen GmbH, Hilden, Germany). All the *p28* and *p25* genes reported in this study were amplified by polymerase chain reaction (PCR). PCR reactions were carried out in 50 μl volume that included 1.5 mM MgCl_2_, 1 X PCR buffer, 1.25 mM of each deoxynucleosidetriphosphate, 0.4 mM of each primer, and 0.03 U/μM of AmpliTaqDNA polymerase (Applied Biosystems, Roche-USA). For the *pvs28* gene, we used the primers 5’-TTTGTTCATTTTTGACATACTCACTT-3’ and 5’-ATGCGCGGTGTGTTATTTGGAG-3’ with an annealing temperature (Ta) of 50°C. For *P*. *cynomolgi*, *P*. *fieldi*, *P*. *fragile*, *P*. *inui* and *P*. *simiovale*, we used the primers 5’-CCAACTGCATTATACAAAAAC-3’ and 5’-ATCTTCTTCGGCGAAAAAA-3’ (Ta: 47°C). For *P*. *knowlesi* and *P*. *hylobati*, we used 5’-TGCCACCCCTTGTTCAAAATG-3’ and 5’-GWACTGACTCTGYGADACC-3’ (Ta: 54°C). In some cases, a nested PCR was required by using the primer sets 5’-ACTTGCTCACTCGACTTAACC-3’ and 5’-CGTTTTTCTTGTCCCTTTGTCAC-3’ (Ta: 53°C) for *P*. *vivax* and 5’-ATACAAAAACGACTCCCCCTTT-3’ and 5’-CGTATGACTTGAACTGACTC-3’ (Ta: 47°C) for NHPPs.

The *pvs25* gene and its orthologs were amplified with the primers 5’-CTGACTTTCGTTTCACAGCA-3’ and 5’-ACATCACAAGTCCGTAAGTT-3’ (Ta: 53°C). In the case of nested PCRs, we combined the external primers 5’-CTGACTTTCGTTTCACAGCA-3’ and 5’-CATCACAAGTCGGTAAGT-3’ (Ta: 53°C) with the internal 5’-TTCGACCGCTCAATTCGCC-3’ and 5’-CAAGTCGGTAAGTTCAGTAAAG-3’ (Ta: 55°C). The amplification conditions for both genes were as follow: 5 min at 95°C, followed by 35 cycles with 1 min of denaturation at 94°C, 1 min at the specific Ta and elongation at 72°C for 2 min. After 35 cycles, a final elongation step at 72°C for 10 min was carried out. Amplified products from the two independents PCRs were either directly purified or gel extracted and cloned in pGEM-T easy Vector Systems I following the manufactory protocol (Promega, USA). For at least two clones, both strands were sequenced using an Applied Biosystems 3730 capillary sequencer. All the sequences reported in this investigation are deposited in the GenBank under the accession numbers KU285229 to KU285332.

### Genetic diversity and natural selection of *pvs28* and *pvs25*

For both genes, *p28* and *p25*, independent alignments of their nucleotide sequences for *P*. *vivax* and their close NHPs malaria species were performed by using the MUSCLE algorithm [25] implemented in SeaView4 [26] on translated sequences followed by visual inspection and manual editing. The protein domains (signal peptide, EGF-like domains and GPI anchor) were assigned in the alignments following the description used by Saxena et al. 2006 [5]. In the case of *pvs28*, the low complexity regions (LCRs) were not included in the polymorphism and phylogenetic analyses; however, those were studied separately as defined by Rich et al. 1997 [27].

We estimated the polymorphism by gene and by domain within each *Plasmodium* species by using the population statistics *π* (the average number of substitutions between any two sequences), number of segregating polymorphic sites (S), and haplotype diversity (Hd). The polymorphism was also explored by computing Tajima’s D statistic [28]. The distribution of the genetic diversity across the *p28* and *p25* gene-sequences was described by calculating *π* on a sliding-window of 50 base pairs (bp) with a step size of 10 sites. The statistic was calculated in each window, assigned to the nucleotide at the midpoint of the window and plotted against the nucleotide position. All these calculations were performed using DnaSP v5.10.01 [29].

Evidence of natural selection was explored by estimating the average number of synonymous substitution per synonymous site (dS) and non-synonymous substitutions per non-synonymous site (dN) between a pair of sequences under the Nei Gojobori method [30], with the Jukes and Cantor corrections as implemented in the MEGA6 [31]. The difference between dS and dN and its standard error was estimated by using bootstrap with 1,000 pseudo-replications, as well as a two tailed codon based Z-test on the difference between dS and dN as described in Nei and Kumar 2000 [32]. Under the neutral model, synonymous substitutions accumulate faster than non-synonymous because they do not affect the parasite fitness and/or purifying selection is expected to act against nonsynonymous substitutions (dS≥dN). Conversely, if positive selection is maintaining polymorphism, a higher incidence of nonsynonymous substitutions is expected (dS<dN). We assumed as a null hypothesis that the observed polymorphism was not under selection (dS = dN).

We also used the random effects likelihood (REL) method as implemented in HyPhy, which uses flexible, but not overly parameter-rich rate distributions [33] and allows both dS and dN to vary across sites independently. REL allows for tests of selection at a single codon site while taking into consideration rate variation across synonymous sites. It is often considered the only method for inferring selection from low divergence alignments such as *pvs28* and *pvs25*. Evidence for natural selection was also explored in *P*. *vivax* by using the McDonald & Kreitman (MK) test which compares intra and inter-specific number of synonymous and non-synonymous changes [34]. In this analysis we compared *P*. *vivax* with their close NHPPs *P*. *cynomolgi*, *P*. *inui* and *P*. *knowlesi* for both *p28* and *p25* genes. Significance was assessed using a Fisher’s exact test for the 2 x 2 contingency table as implemented in the DnaSP.

### Population structure and haplotype network of *pvs28* and *pvs25*

In order to study the genetic relationships among worldwide haplotypes, a median joining network was estimated for a set of 284 cosmopolitan sequences of *pvs28* and 325 of *pvs25* genes by using Network v4.6.1.0 (Fluxus Technologies 2011). Transversions were set equal to transitions and the epsilon parameter set equal to 0 with only one round of star contraction, which collapses star-like structures in the network into single nodes. The total number of sites included in these analyses excluding gaps or missing data were 547 out of 744 for *pvs28* and 558 out of 660 for the *pvs25* genes. In addition, we also used DnaSP to estimate the fixation index (*F*_ST_) based on haplotype-frequencies among these geographical regions.

In order to investigate whether intragenic recombination generates allelic diversity in the *P*. *vivax* ookinete genes, the genetic algorithms for recombination detection (GARD) were used to screen for the recombination breakpoints in both alignments, as implemented in Datamonkey (http://www.datamonkey.org/)[35,36]. Default parameters for the detection of recombination breakpoints and donor-recipient pairs were used with a significance cut-off of 0.05.

### Phylogenetic analyses

The evolutionary relationships among the *p28* and *p25* genes in *Plasmodium* spp. were investigated using Bayesian methods implemented in MrBayes v3.2 with the default priors [37]. A General Time-Reversible model (GTR+I+Γ) was used because it had the lowest likelihood value and possessed the fewest number of parameter that best fit the data (*p28* and *p25*) as was estimated by MEGA6. For both phylogenies (*p28* and *p25*), two independent chains were sampled every 200 generations in runs lasting 6 × 10^6^ Markov Chain Monte Carlo steps, and after convergence was reached, we discarded 50% of the sample as ‘burn-in’ period. Convergence is reached when the value of the potential scale reduction factor is between 1.00 and 1.02 and the average standard deviation of the posterior probability is below 0.01 [37].

Additionally, the adaptive branch-site random effects likelihood (aBSREL) approach [38], implemented in Datamonkey, was run to detect evidence of episodic positive selection on all branches using both phylogenies (*p28* and *p25*). It allows for different Ka/Ks ratios among sites and branches. We performed a likelihood ratio test (LRT) comparing the null model (ω = 1) against the alternative, where the branch was undergoing some form of selection (ω ≠ 1). In addition, we used BUSTED, implemented also in Datamonkey, which is an approach to identify gene-wide evidence of episodic positive selection, where the non-synonymous substitution rate is transiently greater than the synonymous rate [39]. In these analyses we selected both human malarias *P*. *vivax* and *P*. *falciparum* branches because BUSTED requires pre-specified subset of lineages.

## Results

### Evolutionary analyses of *pvs28* and *pvs25*

A total of 284 and 325 sequences were studied for the *pvs28* and *pvs25* genes respectively. Tables 1 and 2 describe the polymorphism found in the complete gene-sequences and their subsequent domains for both genes. The overall genetic diversity, as estimated by π, revealed that both genes are relatively conserved when compared to other vaccine candidates as has been reported by previous studies analyzing a smaller sample size [3,12–17]. The *pvs28* gene showed a slightly higher, but not significant polymorphism level (*π* = 0.0037 ± 0.0011) than *pvs25* (*π* = 0.0023 ± 0.0010). When we compared the polymorphism observed between isolates from Asia and the Americas, we found that *pvs28* samples from Asia were slightly more polymorphic (*π* = 0.0035 ± 0.0012) than the ones from the Americas (*π* = 0.0024 ± 0.0011) (Table 1). In contrast, we observed a similar genetic diversity in the *pvs25* sequences from both Asia (*π* = 0.0017 ± 0.0008) and the Americas (*π* = 0.0018 ± 0.0010) (Table 2). Nevertheless, in both genes the standard errors for *π* overlapped when Asia and the Americas were compared.

**Table 1:** *Pvs28* worldwide polymorphism by gene CDS and gene-domain.

	π (SE)	dS	dN	dS-dN (SD)	*p* (Z-stat)	Tajima’s D (p-value)
**All sequences (N = 284)**						
Gene CDS	0.0037 (0.0011)	0.0014	0.0044	-0.0030 (0.0014)	**0. 0271 (2.2372), dS<dN**	**-2.2857 (p<0.01)**
EGF1	0.0030 (0.0023)	0.0014	0.0034	-0.0020 (0.0032)	0.4975 (0.6805), dS = dN	
EGF2	0.0050 (0.0027)	0.0002	0.0063	-0.0060 (0.0034)	0.0667 (1.8503), dS = dN	
EGF3	0.0059 (0.0030)	0.0036	0.0066	-0.0030 (0.0030)	0.3459 (0.9463), dS = dN	
EGF4	0.0007 (0.0003)	0.0010	0.0006	0.0004 (0.0011)	0.7755 (-0.2858), dS = dN	
GPI anchor	0.0014 (0.0005)	0.0007	0.0018	-0.0011 (0.0008)	0.2261 (1.2167), dS = dN	
**Asia (N = 241)**						
Gene CDS	0.0035 (0.0012)	0.0012	0.0042	-0.0030 (0.0014)	**0.0318 (2.1719), dS = <dN**	**-2.2344 (p<0.01)**
EGF1	0.0033 (0.0025)	0.0016	0.0038	-0.0022 (0.0036)	0.5182 (0.6481), dS = dN	
EGF2	0.0044 (0.0027)	0.0003	0.0056	-0.0053 (0.0037)	0.1373 (1.4960), dS = dN	
EGF3	0.0054 (0.0033)	0.0030	0.0062	-0.0032 (0.0031)	0.3289 (0.9804), dS = dN	
EGF4	0.0005 (0.0003)	0	0.0006	-0.0006 (0.0004)	0.0784 (1.7755), dS = dN	
GPI anchor	0.0017 (0.0006)	0.0008	0.0021	-0.0013 (0.0011)	0.2333 (1.1980), dS = dN	
**America (N = 40)**						
Gene CDS	0.0024 (0.0011)	0.0015	0.0027	-0.0012 (0.0018)	0.4926 (0.6883), dS = dN	-0.6019 (p>0.10)
EGF1	0.0009 (0.0006)	0	0.0011	-0.0011 (0.0008)	0.1754 (1.3633), dS = dN	
EGF2	0.0038 (0.0033)	0	0.0049	-0.0049 (0.0043)	0.2811 (1.0827), dS = dN	
EGF3	0.0054 (0.0041)	0.0037	0.0059	-0.0022 (0.0059)	0.7093 (0.3737), dS = dN	
EGF4	0.0018 (0.0013)	0.0064	0.0008	0.0056 (0.0066)	0.4325 (-0.7876), dS = dN	
GPI anchor	0	0	0	0	1.0 (0.0), dS = dN	

**Table 2:** *Pvs25* worldwide polymorphism by gene CDS and gene-domain.

	π (SE)	Ds	Dn	Ds-Dn (SD.)	*p* (Z-stat)	Tajima’s D (p-value)
**All sequences (N = 325)**						
Gene CDS	0.0023 (0.0010)	0.0007	0.0027	-0.0021 (0.0013)	0.1040 (1.6381), dS = dN	**-2.2461 (p<0.001)**
EGF1	0.0004 (0.0001)	0.0011	0.0003	0.0008 (0.0006)	0.1095 (-1.6126), dS = dN	
EGF2	0.0053(0.0038)	0.0002	0.0067	-0.0067 (0.0047)	0.1631 (1.4034), dS = dN	
EGF3	0.0028 (0.0017)	0.0006	0.0034	-0.0028 (0.0022)	0.2009 (1.2860), dS = dN	
EGF4	0.0009 (0.0003)	0.0017	0.0007	0.0010 (0.0010)	0.3658 (-0.9078), dS = dN	
**Asia (N = 242)**						
Gene CDS	0.0017 (0.0008)	0.0006	0.0020	-0.0014 (0.0011)	0.2241 (1.2221), dS = dN	**-2.3083 (p<0.01)**
EGF1	0.0004 (0.0001)	0.0004	0.0004	0.0000 (0.0004)	0.9870 (0.0163), dS = dN	
EGF2	0.0035 (0.0029)	0	0.0045	-0.0045 (0.0038)	0.2753 (1.0960), dS = dN	
EGF3	0.0019 (0.0014)	0.0006	0.0023	-0.0017 (0.0017)	0.3673 (0.9050), dS = dN	
EGF4	0.0011 (0.0003)	0.0023	0.0009	0.0014 (0.0016)	0.3081 (-1.0235), dS = dN	
**America (N = 83)**						
Gene CDS	0.0018 (0.0010)	0.0009	0.0021	-0.0012 (0.0013)	0.3539 (0.9306), dS = dN	-1.2406 (p>0.10)
EGF1	0.0006 (0.0004)	0.0032	0	0.0032 (0.0020)	0.0711 (-1.8210), dS = dN	
EGF2	0.0037 (0.0035)	0.0008	0.0044	-0.0036 (0.0047)	0.3970 (0.8500), dS = dN	
EGF3	0.0038 (0.0034)	0.0008	0.0048	-0.0039 (0.0046)	0.3467 (0.9098), dS = dN	
EGF4	0.0002 (0.0002)	0	0.0002	-0.0002 (0.0002)	0.2934 (1.0553), dS = dN	

**Fig 1:**
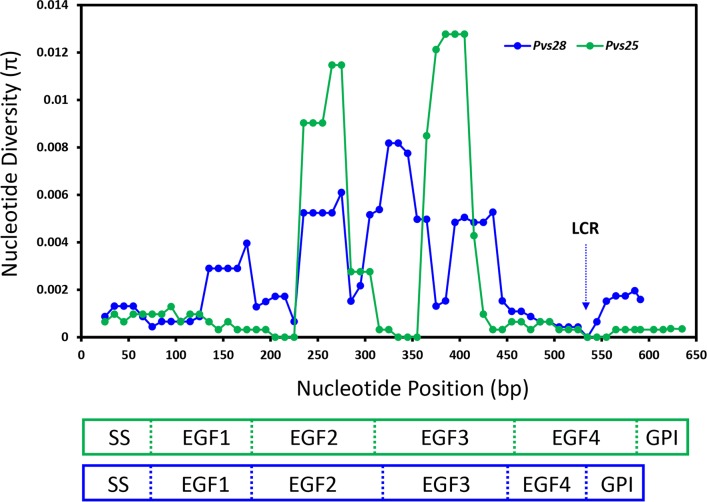
Sliding window analysis of the nucleotide diversity (*π*) on the *pvs28* and *pvs25* genes. Genetic diversity (*π*) was estimated using a sliding window of 50 base pairs (bp) with a step size of 10 sites. The LCR of tandem repeats of the *pvs28* gene was not included but its location is indicated by a blue arrow.

Because of P28 and P25 EGF-like domains carry critical epitopes recognized by *P*. *vivax* TB antibodies [5,8], we also estimated π by gene-domain in addition to the geographic regions. In both genes, the EGF2 and EGF3 were the most polymorphic domains of the proteins (Tables 1 and 2). Fig 1 shows the distribution of the polymorphism across the *pvs28* and *pvs25* genes using a sliding window approach of the nucleotide diversity *π*. The polymorphism for both genes was distributed unevenly; the most conserved areas were located at the secretory signal sequence at the N-terminus, the EGF1 and EGF4 like domains, and the GPI anchor at the C-terminus. In contrast, central regions like the EGF2 and EGF3 domains accumulated higher variability.

We also studied the polymorphism found in *pvs28* (Table 3) and *pvs25* (Table 4) genes by country. Regardless of sampling differences, the haplotype diversity Hd was similar for both genes (*pvs28*-Hd = 0.765, *pvs25*-Hd = 0.724). Yet, the number of haplotypes found in *pvs28* (H = 53) seems to be slightly higher than the ones found in the *pvs25* gene (H = 35). We report estimates by country with at least 10 sequences or more in our alignment. A high haplotype diversity was observed for *pvs28* in Bangladesh (Hd = 0.895), Thailand (Hd = 0.993) for Asia, and Venezuela (Hd = 0.873) from the Americas (Table 3). For *pvs25*, high haplotype diversity was observed in samples from China (Hd = 0.6478), Venezuela (Hd = 0.8000), and Mexico (Hd = 0.6513) (Table 4).

**Table 3:** *Pvs28* gene worldwide polymorphism by countries.

Population	# Seq	Segregating sites (S)	Haplotypes (H)	Haplotype diversity (Hd)	π (SE)	dS	dN	dS-dN (SD)
Mexico	23	2	2	0.474	0.0015 (0.0010)	0	0.0020	-0.0020 (0.0013)
Venezuela	11	6	7	0.873	0.0024 (0.0010)	0.0038	0.0020	0.0018 (0.0031)
China	30	7	6	0.584	0.0033 (0.0014)	0.0011	0.0039	-0.0029 (0.0015)
Korea	93	22	17	0.334	0.0009 (0.0002)	0.0010	0.0008	0.0002 (0.0005)
Thailand	18	15	17	0.993	0.0064 (0.0020)	0.0025	0.0075	-0.0051 (0.0023)*
India	68	13	12	0.437	0.0028 (0.0011)	0	0.0036	-0.0036 (0.0015)**
Bangladesh	20	10	11	0.895	0.0054 (0.0019)	0.0009	0.0067	-0.0058 (0.0026)***
Worldwide	284	51	53	0.765	-	-	-	**-**

**p = 0.0280 (Z-stat = 2.2238)

*p = 0.0090 (Z-stat = 2.6538)

***p = 0.0238 (2.2890)

**Table 4:** *Pvs25* gene worldwide polymorphism by countries.

Population	# Seq	Segregating sites (S)	Haplotypes (H)	Haplotype diversity (Hd)	π (SE)	dS	dN	dS-dN (SD)
China	29	3	4	0.6478	0.0013 (0.0008)	0	0.0016	-0.0016 (0.0011)
India	100	4	6	0.3202	0.0009 (0.0005)	0.0003	0.0011	-0.0008 (0.0007)
Korea	99	24	17	0.5828	0.0017 (0.0008)	0.0010	0.0019	-0.0009 (0.0011)
Mexico	64	3	4	0.6513	0.0012 (0.0008)	0.0002	0.0015	-0.0013 (0.0010)
Venezuela	15	6	7	0.8000	0.0018 (0.0008)	0.0029	0.0016	0.0013 (0.0019)
Worldwide	325	40	35	0.724	**-**	**-**	**-**	**-**

In order to explore how natural selection was involved in the maintenance of the observed polymorphism, we estimated the average number of synonymous (dS) and non-synonymous (dN) changes between two sequences (Tables 1–4). Overall, we found a significant excess of non-synonymous over synonymous polymorphic changes in *pvs28* sequences (*p* = 0.0271, Table 1). Nevertheless, when we estimated the average dS and dN by region, this pattern was maintained in Asia (*p* = 0.0318) but not in the Americas (*p* = 0.4926), specifically in Thailand (*p* = 0.0280), India (*p* = 0.0090), and Bangladesh (*p* = 0.0238). Although there was an excess of non-synonymous (dN = 0.0027) over synonymous substitutions (dS = 0.0007) in *pvs25* polymorphism (Table 2 and Table 4), the differences were not significant (*p* = 0.1040) (Table 2).

We examined the amino acid replacements observed in the P28 and P25 proteins by using *P*. *vivax* Salvador I strain as a reference; our observations are summarized in S1 and S2 Tables respectively. In the Pvs28 protein, we observed 44 amino acid changes, most of them in low frequency (<0.1% on 284 sequences). The EGF1 domain had the lowest number of amino acid replacements with only the replacement 52(M/L) found in 25% (S1 Table). In contrast, the EGF2 domain showed the highest number of changes (12 replacements) but most of them observed in low frequency (<1%). The most frequent replacements were at 65(T/K) found in 21.8% of the sequences, followed by 87(D/N) and 98(L/I) with 8.5% and 4.2% frequency respectively. The EGF3 domain displayed a total of 13 amino acid changes, the most common were in positions 110(N/Y) found in 5.6%, 116(L/V) in 10.9% and 140(T/S) in 16.2% of the total samples. The EGF4 domain (no including LCR of tandem repeats) and the GPI anchor region showed together only 14 changes in low frequency (<3.0%). It is important to emphasize that the polymorphisms in positions 87(D/N) and 110(N/Y) were observed only in the 40 sequences from the Americas with a frequency of 60% and 40% respectively. Here, we report these polymorphisms for the first time in Colombia, El Salvador (Sal II, [40]), Honduras, Nicaragua, Panama and Venezuela. Moreover, some of the frequent amino acid changes observed in Pvs28 (52M/L, 65T/K and 116L/V) were also present in *P*. *cynomolgi* and *P*. *inui* (S1 Table).

**Fig 2:**
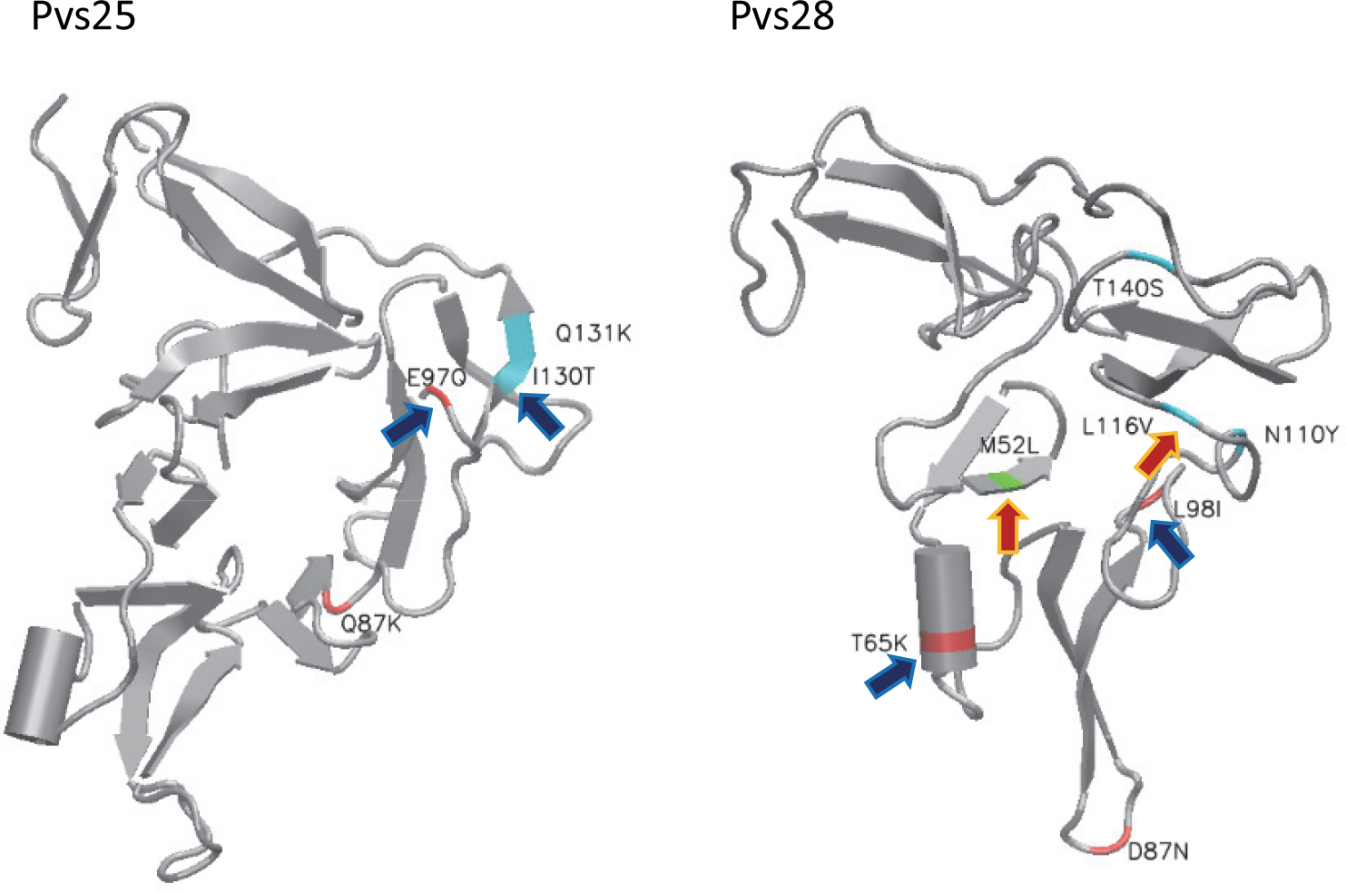
Representation of 3D structures of the Pvs25 and Pvs28 proteins. The mapped amino acid substitutions are those with a frequency >1%. Arrows indicate codons putatively under positive selection under the model implemented in the random effects likelihood (REL) method (blue arrows: Bayes Factor >10<50; red arrows: >50). All substitutions with their frequencies and structural location are listed in S1 and S2 Tables.

A similar analysis for the Pvs25 antigen (n = 325) showed a total of 34 low frequency (<1%) amino acid substitutions (S2 Table). The most frequent changes for the EGF2 were 87(Q/K) found in 12.7% and 97(E/Q) in 50.3% of the worldwide sequences. Glutamine (Q) at position 87 is one of the contacting residues involved in the binding of the transmission blocking antibody 2A8 (Fab VH domain) with the ß loop (EGF2) of the Pvs25 protein [5]. This amino acid was found to be mutated to lysine (K) in several field isolates from Iran, Mauritania, Brazil, Colombia, Mexico and Venezuela. In the EGF3 domain, changes at positions 130(I/T) in 89.1% of the sequences in addition to 131(Q/K) in 8.2% were the most common. In the case of *p25* orthologous genes in close NHP malarias, different amino acid changes were also identified in all these positions (S2 Table). To show the location of the observed mutations on the Pvs28 and Pvs25 structures, the three dimensional structure for Salvador I Pvs28 was modelled by using Phyre2 [41] on the Pvs25 structure as template [5]; 65% of the Pvs28 structure was modelled with 99.4% confidence. Positions of mutations for both Pvs25 and Pvs28 were visualized using Visual Molecular Dynamics (VMD [42]). Mutations with a frequency >1% were mapped by residue location and colored according to domain (Fig 2, S1 and S2 Tables). Residues putatively under positive selection were indicated with arrows (see results from REL method explained later in the text).

### Worldwide *pvs28* and *pvs25* haplotype networks and population structure

**Fig 3:**
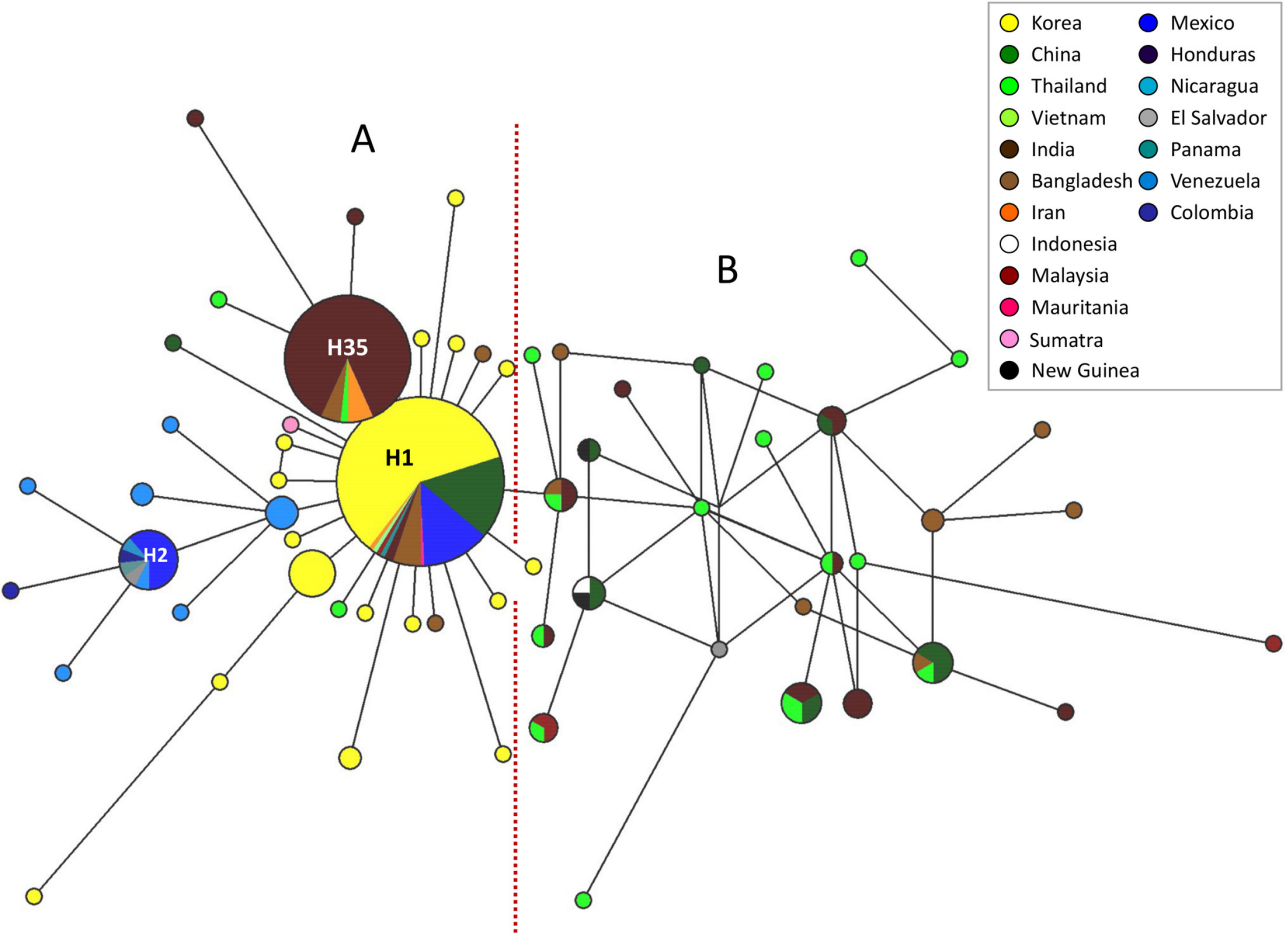
Median joining network of the *P*. *vivax pvs28* haplotypes sampled two or more times plus selected singleton haplotypes. Branch lengths are proportional to divergence; node sizes are proportional to the total haplotype frequencies. The network shows 63 haplotypes found in 284 sequences with a haplotype diversity of 0.765. The most frequent haplotypes are indicated. Every color corresponds to a different geographic origin. Lines separating haplotypes represent mutational steps.

**Fig 4:**
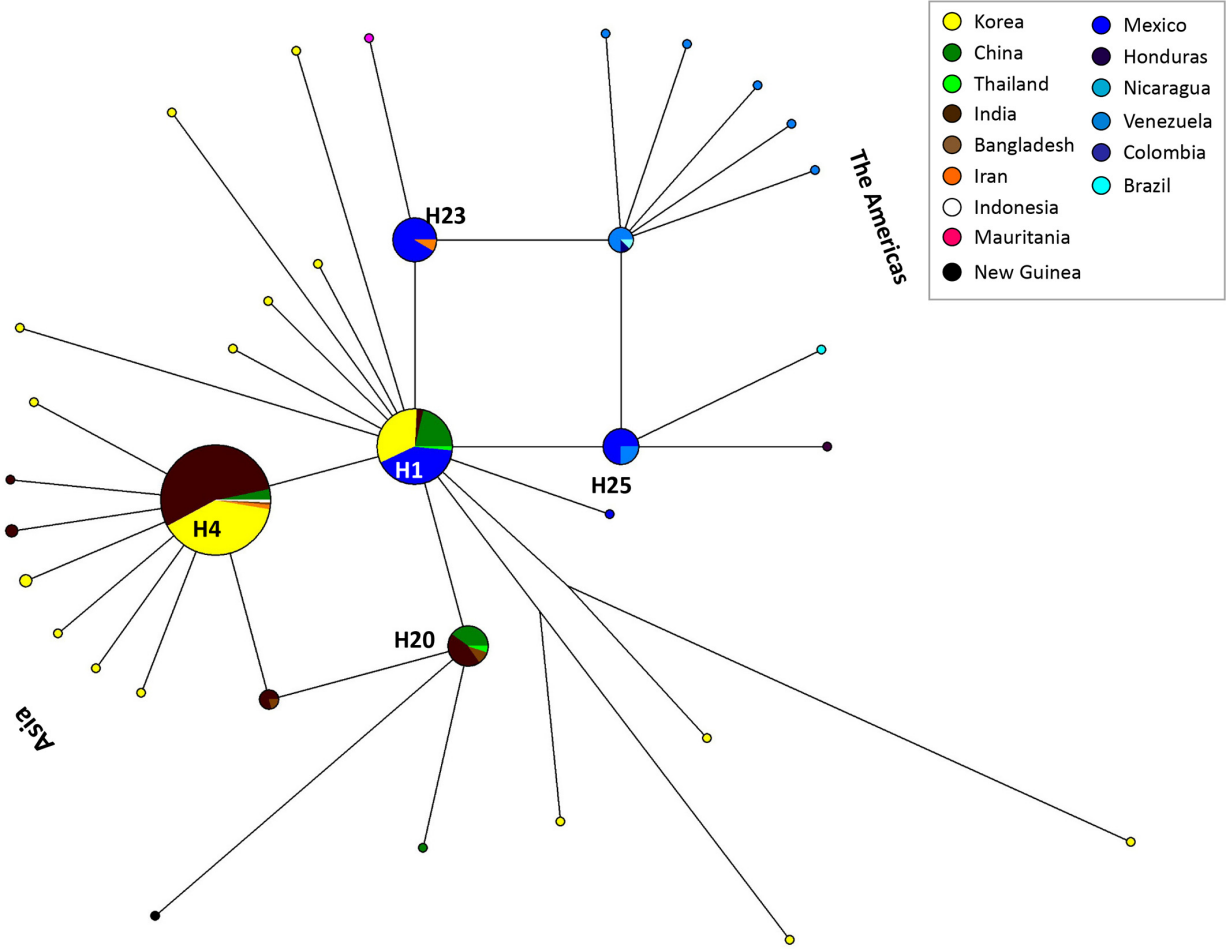
Median joining network of the *P*. *vivax pvs25* haplotypes sampled two or more times plus selected singleton haplotypes. Branch lengths are proportional to divergence; node sizes are proportional to the total haplotype frequencies. The network shows 35 haplotypes found in 325 sequences with an overall haplotype diversity of 0.724. The most frequent haplotypes are indicated. Each color corresponds to a different geographic origin. Lines separating haplotypes represent mutational steps.

The haplotype networks based on the *pvs28* and *pvs25* sequences are shown in Figs 3 and 4 respectively. We identified 63 distinct haplotypes among 284 *pvs28* sequences from 18 regions/countries. Although the sampling effort per country did not allow us to reliably estimate and compare the haplotypes’ relative frequencies, there were some emerging patterns. In particular, we focused on the number of countries/areas where a given haplotype had been found since it is informative of its geographic range.

The *pvs28* network presented two distinctive features referred here to as A and B. The feature A suggests a star-like shape consistent with an expansion of the *P*. *vivax* population for part of the network [18] while the feature B refers to the reticulated structure observed in Asia. Only 12 haplotypes (19.1%) were shared by two countries or more whereas 51 haplotypes (81.0%) were restricted to single countries. Importantly, only three haplotypes were found with a relatively broad distribution (H1, H2, H35; see Fig 3).

The haplotype denominated as H1 was the most frequent (40.1%, 114/284) and showed a worldwide distribution (Fig 3). The haplotype H35 with 59 sequences (20.8%) was the second most predominant and the most common in the Indian samples included in this study (77.3%). It was not only found in five distant Indian regions [17] but also in Bangladesh, China, Iran, and Thailand (Fig 3). The third haplotype in terms of its frequency was H2 (4.6%, 13/284) and belongs to a more divergent cluster which includes only samples from the Americas; specifically, Mexico, Colombia, El Salvador (Sal II strain), Nicaragua, Panama, and Venezuela. The network results suggest that the haplotype H2 could have originated from the most frequent H1 haplotype. Because of the study performed in Korea, which is geographically smaller, involved a large sample collected between 1996 and 2007 [15], we can speculate that haplotype H1 might be the most common in that region. Feature B of the *pvs28* network (Fig 3) showed a group of haplotypes from Bangladesh, China, India, Malaysia, Thailand, and Vietnam forming reticulations. This pattern corresponds to several divergent haplotypes found in very low frequency in this set of samples.

The *pvs25* haplotype network depicts 35 distinct haplotypes among 325 sequences from 15 regions/countries. We found five haplotypes in high frequency (H1, H4, H20, H23, and H25; see Fig 4). The haplotype denominated as H4 corresponded to 152 (46.8%) sequences from Bangladesh, China, India, Indonesia, Iran, Korea, and Thailand. This haplotype is related to H1, the second most predominant with 70 (21.5%) sequences distributed in China, India, Korea, Mexico, and Thailand. The other three haplotypes (H20, H23 and H25) were linked to the most frequent H1 and H4 by long branches. Finally, haplotype 25 was only found in Mexico and Venezuela.

To further determine genetic differentiation among populations, the *F*_*ST*_ fixation index was estimated. Supporting our median joining network results, *F*_*ST*_ values estimated for both genes suggest high genetic differentiation among *P*. *vivax* ookinete genes in different regions (*F*_*ST*_ > 0.15, Tables 5 and 6). Pairwise comparisons between Venezuela and Asia regions (China, Korea, India, Thailand, and Bangladesh), produced high *F*_*ST*_ values for both genes ranging from 0.426 to 0.542 in *pvs28* (Table 5) and from 0.457 to 0.748 for the *pvs25* gene (Table 6). As expected, a similar pattern was observed in pairwise comparisons between Mexico and Asia regions in both genes, suggesting some degree of differentiation between Asia and the Americas populations. However, when Mexico and Venezuela populations were compared, high *F*_*ST*_ values were also observed in the *pvs28* (0.251) and *pvs25* (0.457) coding genes. In contrast, *P*. *vivax* populations from Bangladesh compared to China and Thailand, are consistent with a minimal genetic divergence, suggesting no genetic population structure among these regions for *pvs28* (*F*_*ST*_ <0.05). Tajima’s D produced consistent negative values for both *pvs28* (Table 1) and *pvs25* (Table 2) genes for all populations. In most cases, the results of the test were statistically significant with the exception of the American populations.

**Table 5:** Pairwise *F*_*ST*_ indices of inter-population variance for the *pvs28* gene.

*F*_*ST*_	Mexico	Venezuela	China	Korea	India	Thailand	Bangladesh
**Mexico**							
**Venezuela**	0.251						
**China**	0.273	0.455					
**Korea**	0.241	0.525	0.210				
**India**	0.426	0.542	0.293	0.413			
**Thailand**	0.386	0.477	0.090	0.373	0.292		
**Bangladesh**	0.276	0.426	0.003	0.232	0.212	0.055	

**Table 6:** Pairwise *F*_*ST*_ indices of inter-population variance for the *pvs25* gene.

*F*_*ST*_	China	India	Korea	Mexico	Venezuela
**China**					
**India**	0.452				
**Korea**	0.268	0.080			
**Mexico**	0.245	0.600	0.400		
**Venezuela**	0.634	0.748	0.656	0.457	

In order to investigate if recombination generated allelic diversity, the genetic algorithm recombination detection (GARD [35]) was performed. No evidence of intragenic recombination was detected in these ookinete genes.

### Evolutionary analyses of *p28* and *p25* orthologous genes in NHP malarias

In accordance with previous reports, the amino acid sequence alignments of the P28 and P25 proteins suggest that both are conserved among *Plasmodium* spp. (S1 Fig) [2,5,6,23]. All the *p28* and *p25* sequences included in this study have a conserved hydrophobic signal sequence at the N-terminus (residues 1–23, SignalP 4.1 Server) [43], followed by four cysteine-rich epidermal growth factor EGF-like domains and a short GPI anchor region at the C-terminus (S1 Fig). An invariable number of 20 (~9.7%) and 22 (~10%) cysteine residues were found in all NHPPs P28 and P25 proteins respectively. The EGF4-like domain in P28 proteins contains four rather than six cysteines lacking of the 5–6 disulfide bridge. P28 orthologs showed a high average content of Lys (~7.50%), Leu (~7.23%), Asn (~8.92%), Thr (~7.79%) and Val (~7.47%) (S2A Fig). Likewise, for P25 proteins we found an average content of Glu (~9.25), Gly (~6.86), Lys (~9.67), Leu (~7.54) and Val (~8.32) (S2B Fig).

We compared the polymorphism of *pvs28* and *pvs25* with their orthologs in *P*. *cynomolgi*, *P*. *inui* and *P*. *knowlesi*. S3 and S4 Tables show the genetic variation found in the coding sequence (CDS) and the different domains of *p28* and *p25* genes respectively. We found that *P*. *cynomolgi* (P28-π = 0.0340 ± 0.0049, P25-π = 0.0284 ± 0.0041) and *P*. *inui* (P28-π = 0.0400 ± 0.0045, P25-π = 0.0133 ± 0.0026) had higher genetic polymorphism than their orthologs in *P*. *vivax*. In contrast, the *p28* and *p25* polymorphisms observed in *P*. *knowlesi* (P28-π = 0.0023 ± 0.0011, P25-π = 0.0038 ± 0.0015) were similar to *pvs28* and *pvs25* (Tables 1 and 2). The *P*. *knowlesi* orthologs also have shown no polymorphism in most of the gene domains (S3 and S4 Tables). Although, the *P*. *cynomolgi p28* paralog PCYB_007100 had similar genetic diversity (π = 0.0340 ± 0.0044) to the one considered the *P*. *cynomolgi* ortholog to *pvs28*, PCYB_062530 (π = 0.0340 ± 0.0049), the nucleotide diversity was different across both genes (PCYB_062530 and PCYB_007100, S3B Fig).

We estimated the average pairwise dS and dN for NHPPs orthologs to *p28* and *p25*. In the case of *p28*, especially for *P*. *cynomolgi*, the diversity found in both paralogous genes was biased toward synonymous sites, a pattern consistent with purifying selection (S3 Table). This contrasts with the pattern of positive selection found in *pvs28*. Nevertheless, a similar pattern was found in *P*. *inui*. Although there was an excess of synonymous over non-synonymous polymorphisms in *P*. *cynomolgi* and *P*. *knowlesi p25* CDS, the differences were not significant using the codon based Z-test (S4 Table). Interesting, the dS-dN estimated by domains suggests different patterns of selection acting in the EGF1 (negative selection) and EGF3 (positive selection) like domains in *P*. *cynomolgi* (*p* < 0.05, S4 Table). Again, a similar number of synonymous (dS) and non-synonymous (dN) substitutions was found for *p25* in *P*. *inui* (S4 Table). The assumption of neutrality was further examined in *pvs28* and *pvs25* against their orthologs in *P*. *cynomolgi* and *P*. *inui* by using the MK test. This test showed an excess of nonsynonymous over synonymous polymorphisms in the *pvs25* gene when divergence was compared in *P*. *cynomolgi* and *P*. *inui* (*p* < 0.05, S5 Table). In both cases, the neutrality indexes (NI) were bigger than 1 and the significance of the test was explained by an excess of replacement polymorphisms in *pvs25*. These results suggest a possible pattern of balancing selection since a preponderance of non-synonymous intra-species polymorphisms was observed. Similar trends but no significant departures from neutrality were found for *pvs28* (S5 Table).

**Fig 5:**
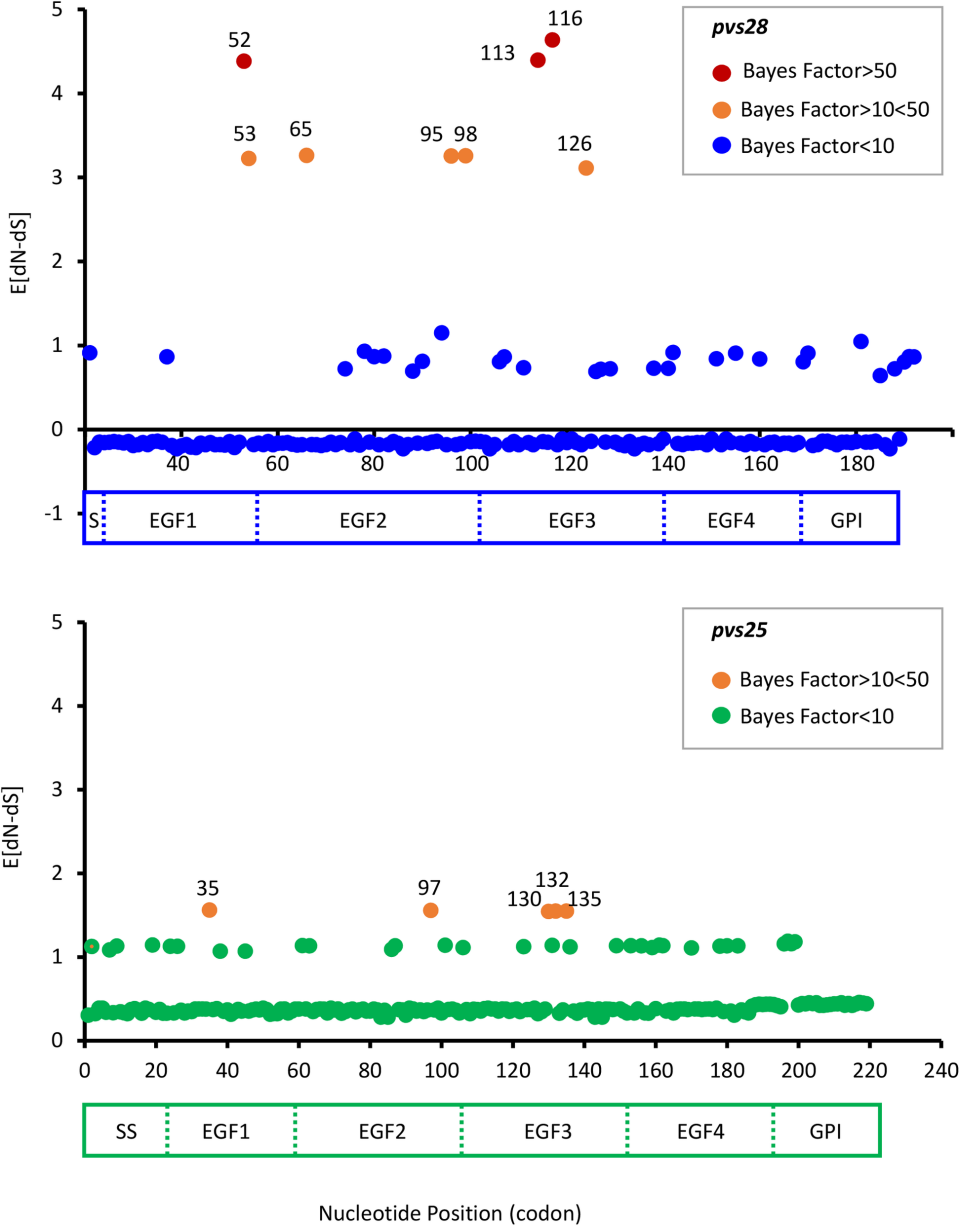
Positively selected codons detected in *pvs28* and *pvs25* using random effects likelihood (REL) method as implemented in HyPhy. The estimated dN-dS (E[dN-dS]) per codon across the *pvs28* and *pvs25* genes are depicted against their position using Salvador I as a reference (see S1 and S2 Tables). Schematic diagrams of Pvs28 and Pvs25 indicate their EGF domains. *Pvs28* codons 52, 113, and 116 yield Bayes factors (BF) of 97.69, 101.51, and 367.92 respectively, indicating that the data provided strong evidence for positive selection. Codons 52, 65, 95, 98, and 123 for Pvs28 and 35, 97, 130, 132, and 135 for *pvs25* have BF higher than 10 but lower than 50, indicating that the data provided some evidence for positive selection acting at those codons. The LCR of *pvs28* was not included in this analysis.

The results of the REL method are depicted in Fig 5 (see S1 Table for reference) and the estimated Bayes Factors (BF) summarize the evidence provided by the data in favor of positive selection. The method detected three codons in *pvs28* where the data provided strong evidence for positive selection with BFs bigger than 50 (codon 52 with a BF of 97.69, codon 113 with a BF of 101.51, and codon 116 with a BF of 367.92; see S1 Table for reference) and five with some evidence for positive selection with BFs bigger than 10 but less than 50 (codons 53, 65, 95, 98, and 123; S1 Table). In the case of *pvs25*, we found only five codons (35, 97, 130, 132, and 135; see S2 Table for reference) where the data provided some evidence of those being under positive selection with BFs bigger than 10 but less than 50 (Fig 5). Residues with mutations in high frequency (>1%) were mapped on the *pvs25* and *pvs28* protein structures depicted in Fig 2.

**Fig 6:**
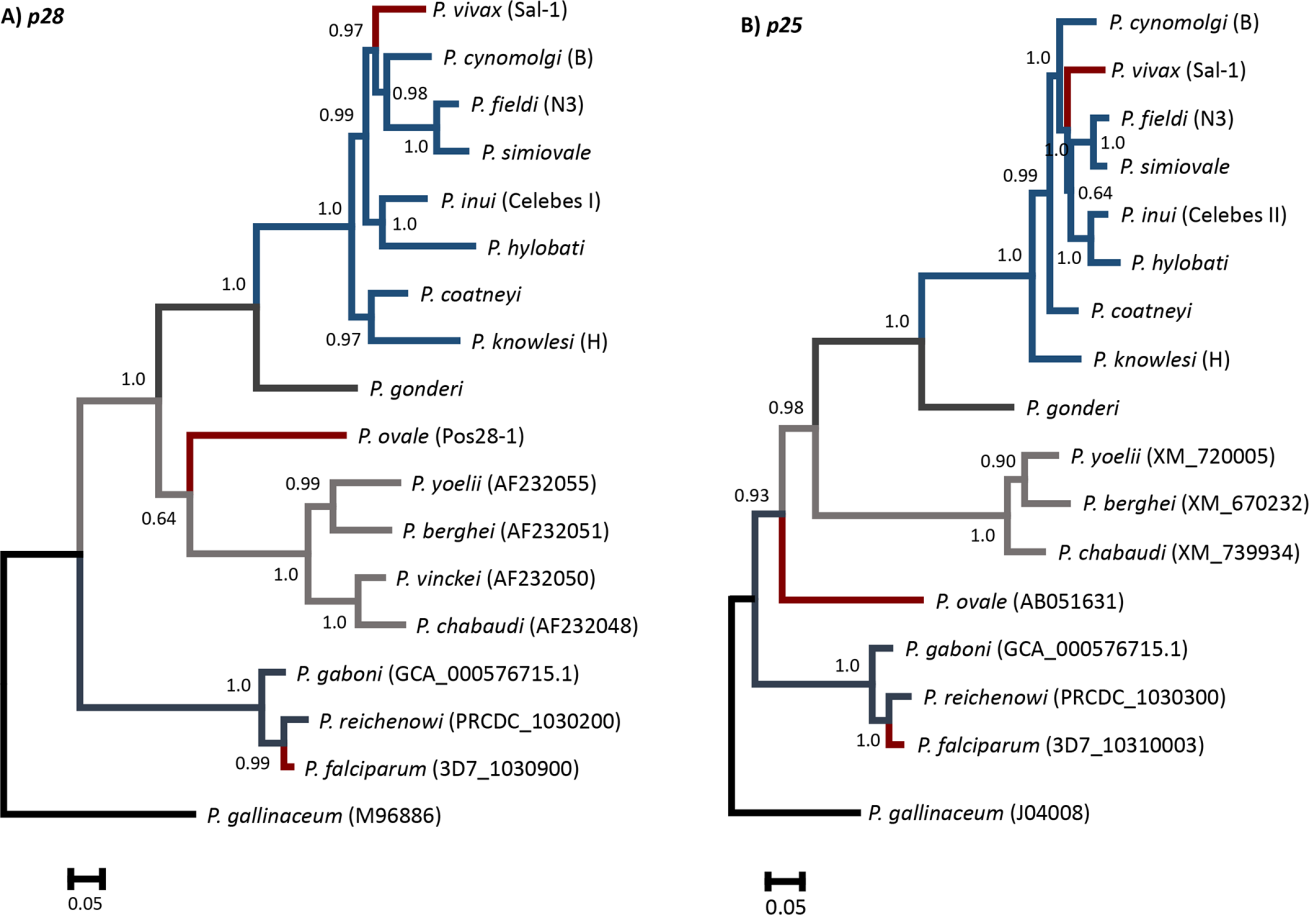
**Bayesian phylogenetic hypothesis constructed with nucleotide sequences of the p28 (A) and p25 (B) genes in Plasmodium spp.** The values above branches are posterior probabilities (see **Methods** section). For both phylogenies (*p28* and *p25*), two independent chains were sampled every 200 generations in runs lasting 6 × 10^6^ Markov Chain Monte Carlo steps.

The Bayesian phylogenies of *p28* and *p25* are depicted in Fig 6. The avian malarial parasite *P*. *gallinaceum* was included as outgroup. Overall, they are comparable with previous published phylogenies using nuclear and mitochondrial genes [19,44–47]. Briefly, in both phylogenies, three major clades were identified: 1) the *Laverania* subgenus, 2) the clade of rodent malarias, and 3) the *P*. *vivax* clade. *Plasmodium vivax* is part of a monophyletic group with closely related NHPPs found in Southeast Asia. The African primate parasite *P*. *gonderi* was consistently placed at the base of this monophyletic group in both phylogenies. The difference between *p28* and *p25* phylogenies was the relative position of *P*. *ovale* (Fig 6). The *p28* phylogeny resembled the phylogenetic tree obtained previously based on the nuclear genes ß-tubulin, CDC-2 and the plastid gene tufA [19].

Two additional phylogenies containing all *p28* and *p25* strains obtained in this study for *P*. *cynomolgi*, *P*. *inui*, and *P*. *knowlesi* were estimated using *P*. *gonderi* as outgroup (S4A and S4B Fig respectively). The three *p28* paralogs found in *P*. *cynomolgi* genome (B strain, PlasmoDB) were included (S4A Fig). The *p28* phylogeny was slightly different to one that included all species; however, the major relationships were maintained (e. g. *P*. *inui*-*P*. *hylobati*, *P*. *fieldi*-*P*. *simiovale* and *P*. *knowlesi*-*P*. *coatneyi*). We could amplify the three different copies only for the *P*. *cynomolgi* strain Berok (S4A Fig), thus we could not confirm that all the *P*. *cynomolgi* strains have the two recent paralogs. Noteworthy, all *p28 P*. *cynomolgi* paralogs formed a monophyletic group that included the ortholog (PCYB-062530) to *pvs25*. This suggests duplication events in *P*. *cynomolgi* that took place after divergence from the common ancestor shared with *P*. *vivax*. To the best of our knowledge, only *P*. *ovale* and *P*. *cynomolgi* have reported paralogs to the *p28* gene. However, we cannot rule out that such duplication events have occurred in others *Plasmodium* spp. In the case of *p25* (S4B Fig), the relationship among NHP malarias from Southeast Asia were the same as those obtained in the phylogeny containing all the species (Fig 6).

Phylogenetic-based methods were used to explore the role that positive selection may have played in the divergence of these two loci. In the case of *p28*, no evidence of episodic diversifying selection was found in any of the 31 total branches using aBSREL (p ≤ 0.05, corrected for multiple testing). However, BUSTED revealed evidence for positive selection acting only on the *P*. *falciparum* lineage (p = 0.002, S6 Table). In contrast, no evidence of episodic diversifying selection was found in the *p25* gene using aBSREL and BUSTED (S6 Table).

### Characterization of the low complexity region (LCR) of the *p28* gene

In order to fully characterize *p28* polymorphism, we examined the LCR of short tandem repeats located at the big C-loop of the EGF4 domain [6]. The description of P28 motifs and amino acid variation is summarized in S7 and S8 Tables. This LCR is also present in all NHPPs that form part of the monophyletic group with *P*. *vivax* including *P*. *gonderi* from Africa and the Pos28-1 of *P*. *ovale* (S7 Table, S1 Fig). In contrast, such LCR is almost absent in species belonging to the *Laverania* clade (*P*. *falciparum* and related species) and rodent malarias with the exception of *P*. *yoelii* (S7 Table, S1 Fig). In the case of *P*. *vivax*, the consensus tandem repeat unit consists of five amino acid motif (GSGGE). The pattern from all *P*. *vivax* sequences can be summarized as [(G/E/S)S(G/R/D)GE]_2–6_, where the first and third positions are polymorphic (S7 and S8 Tables). Interestingly, all the amino acid changes were observed in Asia. The last tandem unit was not a repetitive motif, showing aspartic acid (D) in high frequency at the fifth position, but lower for glutamic acid (E) and glycine (G): [(G/S)S(G/D)G(D/E/G)]. This terminal unit was not included in our polymorphism estimations. Noteworthy, glycine was also the most abundant amino acid in the LCR in all NHP malarias included here (S7 Table).

Since proteins domains containing LCRs might be natural immunogenic carrying possible targets for antigenic epitopes [48], we explored the role of natural selection acting on the observed polymorphism. When the repetitive motifs were aligned among them, a significant (*p* < 0.05) excess of synonymous over non-synonymous substitutions was observed in *P*. *vivax* and NHP malarias (S8 Table), suggesting that the motif is conserved and its sequence might be under purifying selection.

## Discussion

Although *pvs28* shows slightly higher polymorphism than *pvs25*, those differences appear not to be significant. The genetic diversity found in sequences from Asia and the Americas for both genes was similar. This pattern was observed even when there were fewer sequences from the Americas than Asia. This is consistent with studies based on mitochondrial genome sequences (potentially neutral loci) and complete genomes showing that the diversity of *P*. *vivax* population in the Americas is comparable to Asia [18,49].

*Pvs28* and *pvs25* showed higher genetic variability compared to other sexual stage TB antigens reported in *P*. *vivax* as *pvs48/45* (*π* = 0,00053), the Willebrand factor A domain-related protein (WARP) (*π* = 0.00010) and also previous estimations of *pvs25* (*π* = 0.00065) and *pvs28* (*π* = 0.00000) in Korea [50]. The Korean study likely differs from ours because of its limited geographic scope. It is worth noticing that whereas the observed polymorphism is lower than those reported in many merozoite surface antigens such as AMA-1 [51], there are merozoite stage antigens such as MSP-8 and MSP-10 with similar levels of polymorphism to those reported here for the *pvs28* and *pvs25* genes [45].

The neighbor haplotype-network for both *pvs28* and *pvs25* genes formed a star-like shape consistent with the suggested underlying demographic history of a population expansion for *P*. *vivax* [18]. This could also explain the significant and negative Tajima’s D estimated for the gene (Tables 1 and 2). The low global proportion of haplotypes shared between countries for both genes suggests substantial genetic differentiation among *P*. *vivax* populations, as confirmed by high *F*_*ST*_ values (Tables 5 and 6). We also observed some degree of geographic clustering for haplotypes from the Americas; specifically, a divergent clade for the *pvs28* gene characterized by the replacements located at the positions 87(D/N) and 110(N/Y) that were only found in the Americas (S1 Table). Both networks suggest that some of the haplotypes from the Americas could be derived from Asian populations [52,53]; however, the pattern is consistent with previous finding indicating that there was not a recent or single introduction of *P*. *vivax* into the continent [18].

We performed a comparative polymorphism analysis between *pvs25* and *pvs28* and their orthologous genes in the Asian Old World monkey parasites that are closely related: *P*. *cynomolgi*, *P*. *inui* and *P*. *knowlesi*. In contrast to the relatively low genetic diversity observed in *P*. *vivax* and *P*. *knowlesi*, the orthologs in *P*. *cynomolgi* and *P*. *inui* exhibited significantly higher variability. Similar observations have been also reported for genes expressed in asexual *Plasmodium* stages [19,45]. This pattern could be the result of the different demographic histories of these two parasites when compared to *P*. *vivax* and *P*. *knowlesi*. Consistently with the effect of demographic differences, the same pattern has been found in the mtDNA and other genes that are considered neutral [19, 54].

Interestingly, both Pvs28 and Pvs25 proteins showed higher variation at the EGF2 and EGF3 like domains where epitope recognition sites have been identified for blocking antibodies in Pvs25 [5], and predicted for Pvs28 [6] and Pb28 in *P*. *berghei* [55]. Noteworthy, EGF-like domains in the orthologous protein Pfs25 have shown differential immune blocking activity after being separately expressed as a yeast-secreted recombinant protein. In particular, antibodies against the EGF2 domain elicited the strongest blocking activity indicating that this domain might be a good target for TBVs [56].

The EGF-like domains in *Plasmodium* spp. are relatively conserved among genes and closely related species (S1 Fig). A similar pattern has been described in other EGF-like domains expressed in surface proteins from the merozoite, including MSP-4 [57], MSP-5 [58], MSP-8 and MSP-10 [45]. When we estimated the genetic diversity in *P*. *cynomolgi*, *P*. *inui*, and *P*. *vivax*, we observed regions with relatively high polymorphism in EGF2 and EGF3 in both genes (S3 Fig). How this variation affects protein folding and functionality is a matter that remains elusive. However, it has been proposed that EGFs domains can accommodate genetic changes such as gene polymorphism, mutations, insertions and deletions [55]. Consequently, structural folds in the P28 and P25 proteins may not be significantly affected by the observed amino acid changes in natural populations thereby preserving functionality.

Previous investigations suggested that the *p28* and *p25* coding genes were originated as result of a gene duplication event that was prior to the origin of the species included in this investigation [4]. When a duplicated gene neither adapts to a more specialized function nor is silenced by deleterious mutations and continues producing a functional protein, purifying selection could act on both paralogs keeping some level of functional redundancy [59]. Consistently, gene knockouts of either *p25* (P25Sko) or *p28* (P28Sko) alone in *P*. *berghei* have non-significant effects on oocyst production in infected *Anopheles stephensi* mosquitoes. However, concomitant disruption of both genes (Dko) strongly inhibited oocyst production up to 99% [4].

It is worth noting that duplication events have been reported for *p28* in *P*. *ovale* [23] and *P*. *cynomolgi* [24] (confirmed here in the Berok strain, S4 Fig). Furthermore, in the case of *P*. *cynomolgi*, we found evidence of an excess of synonymous over nonsynonymous substitutions in the *p28* paralogous gene PCYB-007100 and PCYB-062530 suggesting purifying selection (S3 Table, S4 Fig). Thus, without evidence indicating pseudogenization and patterns consistent with purifying selection, we can speculate that both *p28* paralogous remain functional in *P*. *cynomolgi*.

We searched for evidence of episodic selection as a consequence of changes in ecology/vectors during the evolution of the species include in this study; however, we did not find it. Only the branch leading to *P*. *falciparum* indicated positive selection in *p28*, a finding that is worth exploring whenever additional *Laverania* species become available. We also explored the effect of selection on the *pvs28* and *pvs25* polymorphisms by performing the MK test and applying codon models such as REL. Their caveat is that these tests usually underperform even when adaptive evolution is present so they are regarded as conservative [60]. The MK test detected evidence for balancing selection in *pvs25*. A similar pattern of synonymous/non-synonymous sites within *P*. *vivax* and its divergence to *P*. *cynomolgi* and *P*. *inui* was found for *pvs28* (see S5 Table), but not significant (p > 0.05). The Bayesian base method (REL), however, detected codons under selection in *pvs28*. In particular, the data provided very strong evidence for selection on three *pvs28* codons with two of those codons, 113 and 116 (Fig 2), yielding BF factors above 100. These two codons are located in the EGF3 domain. We also found other codons in *pvs28* and *pvs25* where the data provided some evidence of those being under positive selection but their BF did not exceed our 50 threshold defined *a priori*. Those residues are indicated with yellow arrows in Fig 2.

The patterns consistent with positive selection acting on the *pvs28* and *pvs25* polymorphism deserve special attention. Whereas the genetic polymorphism observed on surface antigens from the asexual stage has been commonly associated to the selective pressure exerted by the vertebrate immune system [61,62], proteins expressed in the sexual stage may adapt to diverse microenvironments inside *Anopheles* mosquitoes where parasites have to go through in order to complete their life cycle [63]. The fact that anti-Pvs28 and anti-Pvs25 polyclonal antibodies completely block parasite transmission (*Pv*-Sal I) in four species of *Anopheles* mosquitoes [8] indicates that these proteins are essential during this phase of the parasite life-cycle. Whether *pvs25* and *pvs28* facilitate the *Plasmodium* transit through *Anopheles* physical barriers and by so doing, increase the parasite (and may be the vector) fitness is a matter that needs to be investigated [64,65].

The evolutionary and functional implications of LCR in P28 proteins are still elusive. In the case of asexual *Plasmodium* stages, they may have a role interacting with the host adaptive immune system [66,67]. However, such adaptive immune responses are absent in *Anopheles* vectors with the exception of some components from the vertebrate immune system contained in the blood bolus. The P28 LCR has been predicted to be part of a big C-loop, a fast evolving region forming a sheet over the ookinete surface that may affect the binding properties of the protein [6]. Furthermore, other studies have found that terminal LCR, like the one observed in P28, may confer more protein binding capacity [68]. This evidence suggests that the P28 LCR is functionally important. This possibility finds also support on the significant excess of synonymous over nonsynonymous changes on the motifs of most of the NHPPs P28 studied (*p* >0.05) (S7 Table), which indicate evolutionary constrains and not simply conservation from continued homogenization due to gene conversion. Nevertheless, assessing the importance of the LCR on P28 requires experimental evidence that is not currently available.

In summary, we explored the genetic polymorphism of *pvs28* and *pvs25*, and investigated the long term evolution of the genes encoding these antigens within the genus *Plasmodium*. Although they were less diverse than many pre-erythrocytic and erythrocytic stage expressed antigens; their polymorphisms were comparable to others such as MSP-8 and MSP-10. We also found that these genes exhibit comparable diversities in the Americas and in Asia indicating that the use of TBVs against Pvs28 and Pvs25 will likely face similar challenges in both regions. Furthermore, we found two amino acid replacements in Pvs28 (positions 87(D/N) and 110(N/Y)) that appear to be specific for the Americas. Finally, there are polymorphisms that could be maintained by positive selection in both genes and the importance of such observation deserves to be explored. The observation that anti-Pvs28 and anti-Pvs25 polyclonal antibodies can block parasite transmission in some species of *Anopheles* mosquitoes [8] indicates that polymorphism in these proteins could indeed affect the parasite fitness. In particular, *pvs25* and *pvs28* polymorphisms could be the result of differences in vectors acting as selective pressure in some ecological contexts. Consequently, it is possible that a vaccine elicited transmission blocking immune response may not be equally effective across all vector-parasite associations in all epidemiological settings. In this context, exploring the diversity of local alleles and their interactions with specific *Anopheles* species could provide useful information on how to assess TBV efficacy, as well as, how to better deploy these vaccines, even partially effective ones, in the context of malaria control and elimination.

## Supporting Information

S1 Table Pvs28 worldwide amino acids polymorphisms in *P*. *vivax* and closely NHPs malarias. The amino acid variants of the Pvs28 protein were compared against the Salvador I strain (GenBank: AF083503.2) in according to the gene domain and geographic region. Amino acids changes in NHPPs orthologous genes for the correspondent nonsynonymous change in *P*. *vivax* are also showed. LCR of tandem repeats were excluded. [*] denotes new substitutions found to either a specific region or not previously reported. (_&_): Present work; AA: amino acid.
(PDF)

S2 Table Pvs25 worldwide amino acids polymorphisms in *P*. *vivax* and closely NHPs malarias. The amino acid variants of the Pvs25 protein were compared against the Salvador I strain (GenBank: AF083502.1) in according to the gene domain and geographic region. Amino acids changes in NHPP orthologous genes for the correspondent nonsynonymous change in *P*. *vivax* are also showed. LCR of tandem repeats were excluded. [*] denotes new substitutions found to either a specific region or not previously reported. (_&_): Present work; AA: amino acid.
(PDF)

S3 Table *P28* polymorphism by gene CDS and gene-domain in *Plasmodium* spp. (PDF)

S4 Table *P25* polymorphism by gene CDS and gene-domain in *Plasmodium* spp. (PDF)

S5 Table McDonald & Kreitman test for the *pvs28* and *pvs25* genes. (PDF)

S6 Table Likelihood ratio test statistics for Adaptive BSREL and BUSTED analysis of the *p28* and *p25* genes (18 and 17 species respectively). (PDF)

S7 Table Worldwide short tandem repeats in the Pvs28 and NHPPs orthologs. (PDF)

S8 Table Polymorphism in the repetitive motif of the Pvs28 protein and closely NHPPs orthologs. (PDF)

S1 Fig Alignment of the deduced amino acid sequences of the *P28* and *P25* genes. All genes shared a similar gene structure consisting of a signal sequence of 23 amino acids at the N-terminus followed by four cysteine-rich EGF-like domains and a short GPI anchor region at the C-terminus. Cysteines are conserved among *Plasmodium* spp. The species names are abbreviated as follow: Pv, *P*. *vivax*; Pcy, *P*. *cynomolgi*; Pfi, *P*. *fieldi*; Psi, *P*. *simiovale*; Pin, *P*. *inui*; Phy, *P*. *hylobati*; Pco, *P*. *coatneyi*; Pk, *P*. *knowlesi*; Pgo, *P*. *gonderi*; Pov, *P*. *ovale*; Pyo, *P*. *yoelii*; Pbe, *P*. *berghei*; P. vin, *P*. *vinckei*; Pcha, *P*. *chabaudi*; Pga, *P*. *gallinaceum*; Pre, *P*. *reichenowi*; Pf, *P*. *falciparum*, and Pga, *P*. *gaboni*.
(PDF)

S2 Fig **Amino acid composition of the P28 (A) and P25 (B) proteins for *P*. *vivax* and closely NHP malarias.**
(PDF)

S3 Fig **Sliding window analysis of the nucleotide diversity (*π*) of the *p28* gene (A), *P*. *cynomolgi p28* paralogous genes (B), and the *p25* gene (C).** The genetic diversity was estimated by calculating (*π*) on a window of 50 base pairs moving it in steps of 10 sites. LCR were not included.
(PDF)

S4 Fig **Bayesian phylogenetic hypothesis constructed using nucleotide sequences of the genes encoding the P28 (A) and P25 (B) antigens from *Plasmodium* spp. and strains amplified in this study.** The values above branches are posterior probabilities (see Methods section). For both phylogenies (*p28* and *p25*), two independent chains were sampled every 200 generations in runs lasting 6 × 10^6^ Markov Chain Monte Carlo steps. In the case of P28 (A), the clade of *P*. *cynomolgi* consists of three subgroups (1–3). “1” refers to the lineage “PCYB-062530”, which includes strains of its orthologous gene (Ceylonensi, Smithsonian, Gombak). The “2” and “3” subgroups refer to the lineages “PCYB-007100” (Mulligan, PT-1, BX-20, RO) and “PCYB-062510” (Cambodia) respectively, which contain strains corresponding to its paralogous genes.
(PDF)
