# Supplementary material for: Evolution of the Transmission-Blocking Vaccine Candidates Pvs28 and Pvs25 in Plasmodium vivax: Geographic Differentiation and Evidence of Positive Selection
Source: PLoS Negl Trop Dis. 2016 Jun 27;10(6):e0004786. doi: 10.1371/journal.pntd.0004786 (PMC4922550; doi:10.1371/journal.pntd.0004786)
Supplement: S1 Table — The amino acid variants of the Pvs28 protein were compared against the Salvador I strain (GenBank: AF083503.2) in according to the gene domain and geographic region. Amino acids changes in NHPPs orthologous genes for the correspondent nonsynonymous change in P. vivax are also showed. LCR of tandem repeats were excluded. [*] denotes new substitutions found to either a specific region or not previously reported. (&): Present work; AA: amino acid. (PDF) [file pntd.0004786.s001.pdf]

S1 Table. Pvs28 worldwide amino acids polymorphisms in *P. vivax* and closely NHPs malarias. The amino acid variants of the Pvs28 protein were compared against the Salvador I strain (Gen Bank: AF083503.2) in according to the gene domain and geographic region. Amino acids changes in NHPPs orthologous genes for the correspondent nonsynonymous change in *P. vivax* are also showed. LCR of tandem repeats were excluded. [*] denotes new substitutions found to either a specific region or not previously reported. (&): Present study; AA: amino acid.

P28		Domain	SS						EGF1			EGF2											EGF3												EGF4				GPI									
		AA Position	5	14	15	16	18	20	36	52	53	65	73	77	79	81	87	89	93	95	98	105	106	110	113	116	123	125	126	128	137	140	141	150	154	159	168	169	180	184	187	189	190	191	192	203	Ref
P. vivax		Salvador I	H	V	L	T	K	T	V	M	A	T	N	Y	V	A	D	E	R	G	L	E	V	N	N	L	I	D	P	N	N	T	T	K	T	K	E	T	G	S	N	S	A	V	I	M	
		P vivax frequency (%)	< 0,1	< 0,1	< 0,1	< 0,1	< 0,1	< 0,1	< 0,1	25,0 (M/L)	< 0,1	21,8 (T/K)	< 0,1	< 0,1	< 0,1	< 0,1	8,4 (D/N)	< 0,1	< 0,1	< 0,1	4,2 (L/I)	< 0,1	< 0,1	5,6 (N/Y)	< 0,1	10,9 (L/V)	< 0,1	< 0,1	< 0,1	< 0,1	< 0,1	16,1 (T/S)	< 0,1	< 0,1	< 3	< 3	< 3	< 3	< 3	< 3	< 3	< 3	< 3	< 3	< 3	< 3	
	America	Colombia	-	-	-	-	-	-	-	L*	-	-	-	-	-	-	N*	-	-	-	-	-	-	Y*	-	-	-	-	-	-	-	S*	-	-	-	-	-	-	-	-	-	-	-	-	-	-	[&]
		El Salvador [Sal-II]	-	-	-	-	-	-	-	L*	-	-	-	-	-	-	N*	-	-	-	-	-	-	Y*	-	-	-	-	-	-	-	S*	-	-	-	-	-	-	-	-	-	-	-	-	-	-	[&]
		Honduras	-	-	-	-	-	-	-	L*	-	-	-	-	-	-	N*	-	-	-	-	-	-	Y*	-	-	-	-	-	-	-	S*	-	R*	-	-	-	-	-	-	-	-	-	-	-	-	[&]
		Nicaragua	-	-	-	-	-	-	-	L*	-	-	-	-	-	-	N*	-	-	-	-	-	-	Y*	-	-	-	-	-	-	-	S*	-	-	-	-	-	-	-	-	-	-	-	-	-	-	[&]
		Mexico	-	-	-	-	-	-	-	L	-	-	-	-	-	-	N	-	-	-	-	-	-	Y	-	-	-	-	-	-	-	S	-	-	-	-	-	-	-	-	-	-	-	-	-	-	[14]
		Panama	-	-	-	-	-	-	-	L*	-	-	-	-	-	-	N*	-	-	-	-	-	-	Y*	-	-	-	-	-	-	-	S*	-	-	-	-	-	-	-	-	-	-	-	-	-	-	[&]
		Venezuela	-	-	-	-	-	-	A*	L*	-	-	-	-	-	-	N*	G*	-	-	-	-	-	Y*	-	-	-	-	-	-	-	S*	-	-	A*	-	-	-	-	-	-	-	-	-	-	-	[&]
	Asia	Bangladesh	-	-	-	-	-	-	-	L	-	K	-	-	-	-	-	-	-	-	I	-	-	-	S	V	-	-	-	-	-	S	-	-	-	R	-	-	-	-	D	-	-	-	M	-	[20]
		China	-	L	-	-	-	-	-	L	-	-	-	-	-	-	-	-	-	-	I	K	-	-	-	V	-	-	-	-	-	S	-	-	-	-	-	-	-	-	-	-	-	-	M	-	[13]
		India	Y/T	-	-	-	-	-	-	L	V	K	-	-	E	-	-	-	-	-	I	K	-	-	-	V	-	-	-	-	-	S	-	-	-	-	-	-	R	T	-	-	-	L	M	-	[16]
		Iran	-	-	-	-	-	-	-	L	-	K	-	-	-	-	-	-	-	-	-	-	-	-	-	-	-	-	-	-	-	S	-	-	-	-	-	-	-	-	-	-	-	-	-	-	[17]
		Korea	-	-	P	I	M	I	-	L	-	-	D	H	-	T	-	-	Q	-	F	-	-	-	-	-	L/V	-	S	H	Y	S	S	-	-	-	-	A	R	-	-	Y	P	-	-	-	[15]
		Malaysia	-	-	-	-	-	-	-	L*	V*	-	-	-	-	-	-	-	-	-	-	-	E*	-	K*	A*	-	-	-	-	-	S*	-	-	-	-	-	-	-	-	-	-	-	-	M*	-	[&]
		New Guinea	-	-	-	-	-	-	-	L*	-	-	-	-	-	-	-	-	-	-	-	-	-	-	-	-	-	-	-	-	-	-	-	-	-	-	-	-	-	-	-	-	-	-	-	-	[&]
		Thailand	-	-	-	-	-	-	-	L	V	K/I*	-	-	-	V	-	-	-	N	I	K	E	-	-	V*	-	N	-	-	-	S	-	-	-	R*	D	-	-	-	-	-	-	-	-	V*	[12][&]
		Vietnam	-	-	-	-	-	-	-	L*	-	-	-	-	-	-	-	-	-	-	-	-	-	-	-	-	-	-	-	-	-	S	-	-	-	-	-	-	-	-	-	-	-	-	-	-	[&]
	Africa	Mauritania	-	-	-	-	-	-	-	L*	-	-	-	-	-	-	-	-	-	-	-	-	-	-	-	-	-	-	-	-	-	S*	-	-	-	-	-	-	-	-	-	-	-	-	-	-	[&]
P. cynomolgi	Asia	Sri Lanka	Q*	-	-	A*	N*	-	-	L*	-	E*	S*	-	-	T*	V*	-	L*	I*	E*	D*	-	-	-	V*	-	-	-	-	D*	-	-	-	-	-	L*	-	-	-	-	-	-	-	-	-	[&]
		Malaysia	Q*	-	-	A*	N*	-	-	L*	-	E*	S*	-	L*/A*	T*	V*	-	L*	I*	E*	D*	-	-	K*/R*	V*	-	-	-	-	D*	-	-	-	-	-	L*	-	-	-	-	-	-	-	-	I*	[&]
P. inui		India	-	-	-	A*	-	-	-	L*	-	K*	-	-	-	I*	-	-	Q*	T*	T*	N*	-	A*	S*	V*	-	-	-	-	D*	-	-	-	-	-	L*	-	-	-	-	Y*	S*	-	-	-	[&]
		Indonesia	-	-	-	A*	-	-	-	L*	-	K*	S*	-	-	I*	-	-	Q*	T*	T*	D*	-	A*	S*	A*	-	-	-	-	D*	-	-	-	-	-	L*	-	-	-	-	Y*	S*	-	-	-	[&]
		Malaysia	-	-	-	A*	-	-	-	L*	-	K*	-	-	-	I*	-	-	N*/Q*/H*	I*/T*	T*/R*	T*/N*/S*	-	A*/D*	T*/D*/S*	V*	-	-	-	D*	D*	-	-	-	I*	-	L*	-	-	-	-	Y*	S*	-	-	T*	[&]
		Philippines	-	-	-	A*	-	-	-	L*	-	K*	-	-	-	I*	-	-	Q*	T*	T*	N*	-	E*	S*	V*	-	-	-	-	D*	-	-	-	-	-	L*	-	-	-	-	Y*	S*	-	-	-	[&]
		Taiwan	-	-	-	A*	-	-	-	L*	-	K*	-	-	-	I*	-	-	N*	T*	T*	T*	-	A*/T*	T*	-	-	-	-	-	D*	-	-	-	-	-	L*	-	-	-	-	Y*	S*	-	-	-	[&]
P. knowlesi		Malaysia	-	-	-	A*	-	-	I*	V*	T*	K*	P*	-	-	T*	D*	Q*	L*	T*	P*	N*	-	R*	D*	I*	M*	-	-	-	D*	-	V*	-	-	-	L*	-	-	-	-	-	-	-	-	-	[&]
P. coatneyi			-	-	-	A*	-	-	I*	K*	T*	K*	P*	-	-	T*	-	Q*	L*	T*	T*	D*	-	R*	-	E*	-	-	-	-	-	-	I	-	-	-	L*	-	-	-	-	-	-	-	-	-	[&]
P. fieldi			-	-	-	A*	-	-	I*	-	K*	S*	P*	-	M*	V*	H*	G*	L*	I*	E*	N*	-	N*	-	S*	-	S*	-	-	D*	-	I*	E*	-	-	L*	-	-	-	-	-	-	-	-	-	[&]
P. hylobati			-	-	-	A*	N*	-	-	-	E*	A*	P*	-	-	T*	G*	-	-	T*	T*	N*	-	T*	Y*	E*	V*	-	-	S*	-	I*	-	-	-	-	L*	-	-	G*	-	-	S	-	-	-	[&]
P. simiovale			N*	-	-	A*	-	-	I*	-	K*	-	P*	-	A*	V*	H*	G*	L*	I*	E*	N*	-	N*	-	S*	-	S	-	-	D*	-	V*	Q*	-	-	L*	-	A*	-	-	-	-	-	-	-	[&]
Location									β1	β3	β3	#	#	#	β5	β5	#	#	β6	β6	#	#	#	η2	#	#	β9	β9	β9	#	#	β11	β11	β12	#	#	β1	β1									
