# Supplementary material for: Evolution of the Transmission-Blocking Vaccine Candidates Pvs28 and Pvs25 in Plasmodium vivax: Geographic Differentiation and Evidence of Positive Selection
Source: PLoS Negl Trop Dis. 2016 Jun 27;10(6):e0004786. doi: 10.1371/journal.pntd.0004786 (PMC4922550; doi:10.1371/journal.pntd.0004786)
Supplement: S2 Table — The amino acid variants of the Pvs25 protein were compared against the Salvador I strain (GenBank: AF083502.1) in according to the gene domain and geographic region. Amino acids changes in NHPP orthologous genes for the correspondent nonsynonymous change in P. vivax are also showed. LCR of tandem repeats were excluded. [*] denotes new substitutions found to either a specific region or not previously reported. (&): Present work; AA: amino acid. (PDF) [file pntd.0004786.s002.pdf]

S2 Table. Pvs25 worldwide amino acids polymorphisms in *P. vivax* and closely NHPs malarias. The amino acid variants of the Pvs25 protein were compared against the Salvador I strain (GenBank: AF083502.1) in according to the gene domain and geographic region. Amino acids changes in NHPPs orthologous genes for the correspondent nonsynonymous change in *P. vivax* are also showed. LCR of tandem repeats were excluded. [*] denotes new substitutions found to either a specific region or not previously reported. (&): Present study; AA: amino acid.

P25		Domain	SS		EGF1			EGF2							EGF3								EGF4											GPI	
		AA Position	2	19	26	35	45	61	63	86	87	97	101	106	123	130	131	132	135	136	149	153	156	159	161	162	170	178	180	183	196	197	198	199	Ref
P. vivax		Salvador I	N	Y	V	L	M	K	E	A	Q	E	L	C	I	I	Q	S	C	S	K	K	E	C	L	K	C	K	Q	E	S	Y	S	V	
		P. vivax frequency (%)	< 0,1	< 0,1	< 0,1	< 0,1	< 0,1	< 0,1	< 0,1	< 0,1	12,7 (Q/K)	50,3 (E/Q)	< 0,1	< 0,1	< 0,1	89,0 (I/T)	8,1 (Q/K)	< 0,1	< 0,1	< 0,1	< 0,1	< 0,1	< 0,1	< 0,1	< 0,1	< 0,1	< 0,1	< 0,1	< 0,1	< 0,1	< 0,1	< 0,1	< 0,1	< 0,1	
	America	Brazil	-	-	-	-	-	-	-	-	K*	-	-	-	-	-	-	-	-	-	-	-	-	-	-	-	-	-	-	-	-	-	-	-	[&]
		Colombia	-	-	-	-	-	-	-	-	K*	-	-	-	-	-	-	-	-	-	-	-	-	-	-	-	-	-	-	-	-	-	-	-	[&]
		Honduras	-	-	-	-	-	-	-	-	-	-	-	-	-	-	-	-	-	-	-	-	-	-	-	-	-	-	-	-	-	-	-	-	[&]
		Nicaragua	-	-	-	-	-	-	-	-	-	-	-	-	-	-	-	-	-	-	-	-	-	-	-	-	R*	-	-	-	-	-	-	-	[&]
		Mexico	-	-	-	-	-	-	-	-	K	-	-	-	-	T	-	-	-	-	-	-	-	-	-	-	-	-	-	-	-	-	-	-	[14]
		Venezuela	-	-	-	-	-	-	-	-	K*	-	-	-	-	-	-	-	R*	P*	-	-	-	-	-	-	-	-	-	-	-	C*	-	-	[&]
	Asia	Bangladesh	-	-	-	-	-	-	-	-	-	Q	-	-	-	T	K	-	-	-	-	-	-	-	-	-	-	-	-	-	-	-	-	-	[20]
		China	-	-	-	M	-	-	-	-	-	Q	-	-	-	T	K	-	-	-	-	-	-	-	-	-	-	-	-	-	-	-	-	-	[13]
		India	-	-	-	-	-	-	-	-	-	Q	-	-	-	T	K	-	-	-	-	-	-	-	-	-	-	-	-	K	F	-	T	E	[16]
		Iran	-	-	-	-	-	-	-	-	K	Q	-	-	-	T	-	-	-	-	-	-	-	-	-	-	-	-	-	-	-	-	-	-	[17]
		Indonesia	-	-	-	-	-	-	-	-	-	Q	-	-	-	T		-	-	-	-	-	-	-	-	-	-	-	-	-	-	-	-	-	[&]
		Korea	D	-	A	P	T	Q	K	P	-	Q	S	R	F	T	-	-	G	-	-	R	D	R	W	R	-	R	E	-	-	-	-	-	[&]
		New Guinea	-	-	-	-	-	-	-	-	-	-	-	-	-	T	K*	R*	-	-	N*	-	-	-	-	-	-	-	-	-	-	-	-	-	[&]
		Tailandia	-	-	-	-	-	-	-	-	-	Q	-	-	-	T	K	-	-	-	-	-	-	-	-	-	-	-	-	-	-	-	-	-	[12]
	Africa	Mauritania	-	C*	-	-	-	-	-	-	K*	-	-	-	-	T*	-	-	-	-	-	-	-	-	-	-	-	-	-	-	-	-	-	-	[&]
P. cynomolgi	Asia	Cambodia	-	-	-	-	I*	-	-	E*	R*	-	-	-	-	V*	-	-	-	-	-	-	-	-	-	-	-	-	-	-	-	-	-	-	[&]
		India	-	-	-	-	I*	-	-	E*	R*	-	-	R*	-	V*	-	N*	-	-	Q*	-	-	-	-	-	-	-	-	-	-	-	-	-	[&]
		Malaysia	-	-	-	-	I*	-	-	E*	-	-	-	-	-	V*	-	-	-	-	Q*	-	-	-	-	-	-	-	-	-	-	-	-	-	[&]
		Sry Lanka	-	-	-	-	T*	-	-	E*	R*	-	-	-	-	V*	-	-	-	-	-	-	-	-	-	-	-	-	-	-	-	-	-	-	[&]
P. inui		Indonesia	-	-	-	-	I*	-	-	G*	-	-	M*	-	-	V*	-	-	-	-	-	-	-	-	-	-	-	-	-	-	-	-	-	-	[&]
		Malaysia	-	-	-	-	I*	-	-	G*	-	-	M*	-	-	V*	-	-	-	-	-	-	-	-	-	-	-	-	-	-	-	-	-	-	[&]
		Taiwan	-	-	-	-	I*	-	-	G*	-	-	M*	-	-	V*	-	-	-	-	-	-	-	-	-	-	-	-	-	-	-	-	-	-	[&]
P. knowlesi		Philipines	-	-	-	-	I*	-	-	-	-	K*	-	-	-	V*	K*	-	-	-	-	-	-	-	-	-	-	-	-	-	-	N*	-	-	[&]
		Malaysia	-	-	-	-	I*	-	-	-	-	K*	-	-	-	V*	K*	-	-	-	-	-	-	-	-	-	-	-	-	-	-	N*	-	-	[&]
P. coatneyi		Malaysia	-	-	-	-	I*	-	-	-	-	-	-	-	-	V*	K*	-	-	-	-	-	-	-	-	-	-	-	-	-	-	-	-	-	[&]
P: fieldi		Malaysia	-	-	-	-	I*	-	-	P*	-	D*	-	-	-	V*	-	-	-	-	-	-	-	-	-	-	-	-	-	-	-	H*	-	-	[&]
P hylobati		Malaysia	-	-	-	-	I*	-	-	G*	-	-	M*	-	-	V*	-	-	-	-	-	-	-	-	-	-	-	-	-	-	-	-	-	-	[&]
P. simiovale		Sri Lanka	-	-	-	-	I*	-	-	P*	-	D*	-	-	-	V*	-	-	-	-	-	-	-	-	-	-	-	-	-	-	-	-	-	-	[&]
Location					#	β1	β2	#	#	#	#	#	β7	#	β9	β10	β10	β10	β10	β10	#	β12	#	#	#	#	β13	β14	β14	#					
