# Supplementary material for: Evolution of the Transmission-Blocking Vaccine Candidates Pvs28 and Pvs25 in Plasmodium vivax: Geographic Differentiation and Evidence of Positive Selection
Source: PLoS Negl Trop Dis. 2016 Jun 27;10(6):e0004786. doi: 10.1371/journal.pntd.0004786 (PMC4922550; doi:10.1371/journal.pntd.0004786)
Supplement: S3 Table — (PDF) [file pntd.0004786.s003.pdf]

S3 Table. ***P28*** **polymorphism by gene CDS and gene-domain in** ***Plasmodium*** **spp.**

	π (SE)[c]	dS	dN	dS-dN (SD.)	*p* (Z-stat)
P. cynomolgi **Gene PCYB_007100 (N=7)**					
Gene CDS	0.0340 (0.0044)	0.0694	0.0213	0.0482 (0.0172)	**0.0077 (-2.7087), dS>dN**
EGF1	0.0201 (0.0091)	0.1192	0	0.1192 (0.0610)	**0.0442 (-2.0331), dS>dN**
EGF2	0.0304 (0.0089)	0.0158	0.0357	-0.0199 (0.0203)	0.3527 (0.9330), dS=dN
EGF3	0.0185 (0.0078)	0.0401	0.0133	0.0268 (0.0328)	0.3742 (-0.8919), dS=dN
EGF4	0.0470 (0.0159)	0.1946	0.0196	0.1750 (0.1091)	0.0921 (-1.6982), dS=dN
GPI anchor	0.0306 (0.0102)	0.0735	0.0191	0.0544 (0.0438)	0.1979 (-1.2948), dS=dN
P. cynomolgi **Gene PCYB_062530 (N=4)**					
Gene CDS	0.0340 (0.0049)	0.0680	0.0264	0.0416 (0.0158)	**0.0113 (-2.5739), dS>dN**
EGF1	0.0088 (0.0058)	0.0485	0	0.0485 (0.0363)	0.1764 (-1.3601), dS=dN
EGF2	0.0324 (0.0101)	0.0181	0.0371	-0.0190 (0.0234)	0.4533 (0.7524), dS=dN
EGF3	0.0362 (0.0117)	0.0380	0.0370	0.0010 (0.0317)	0.9776 (-0.0281), dS=dN
EGF4	0.0329 (0.0146)	0.1096	0.0182	0.0915 (0.0709)	0.1952 (-1.3026), dS=dN
GPI anchor	0.0479 (0.0163)	0.0788	0.0435	0.0353 (0.0478)	0.4812 (-0.7065), dS=dN
P. inui **(N=12)**					
Gene CDS	0.0400 (0.0045)	0.0378	0.0426	-0.0049 (0.0110)	0.6715 (0.4251), dS=dN
EGF1	0.0373 (0.0115)	0.0469	0.0366	-0.0103 (0.0333)	0.7745 (-0.2872), dS=dN
EGF2	0.0677 (0.0114)	0.0265	0.0845	-0.0580 (0.0200)	**0.0078 (2.7067), dS<dN**
EGF3	0.0535 (0.0122)	0.0396	0.0618	-0.0222 (0.0288)	0.4625 (0.7371), dS=dN
EGF4	0.0161 (0.0060)	0.0157	0.0167	0.0010 (0.0097)	0.9165 (0.1050), dS=dN
GPI anchor	0.0136 (0.0069)	0	0.0190	-0.0190 (0.0098)	**0.0451 (2.0248), dS<dN**
P. knowlesi **(N=5)**					

Gene CDS	0.0023 (0.0011)	0.0055	0.0015	0.0040 (0.0041)	0.3191 (-1.0005), dS=dN
EGF1	0	0	0	0	1.0 (0.0), dS=dN
EGF2	0	0	0	0	1.0 (0.0), dS=dN
EGF3	0.0031 (0.0031)	0.0146	0	0.0146 (0.0158)	0.3337 (-0.9705), dS=dN
EGF4	0	0	0	0	1.0 (0.0), dS=dN
GPI anchor	0.0046 (0.0044)	0	0.0064	-0.0064 (0.0063)	0.3299 (0.9782), dS=dN

[a] PCYB_007100 is paralog to PVX_111180 (strain Salvador I)
[b] PCYB_062530 is orthologue to PVX_111180 (strain Salvador I)
[c] (SE) Standard error
