# Supplementary material for: Evolution of the Transmission-Blocking Vaccine Candidates Pvs28 and Pvs25 in Plasmodium vivax: Geographic Differentiation and Evidence of Positive Selection
Source: PLoS Negl Trop Dis. 2016 Jun 27;10(6):e0004786. doi: 10.1371/journal.pntd.0004786 (PMC4922550; doi:10.1371/journal.pntd.0004786)
Supplement: S4 Table — (PDF) [file pntd.0004786.s004.pdf]

S4 Table. *P25* polymorphism by gene CDS and gene-domain in *Plasmodium* spp.

	**π (SE)[a]**	**dS**	**dN**	**Ds-Dn (SD.)**	***p* (Z-stat)**
***P. cynomolgi* (N=9)**					
Gene CDS	0.0284 (0.0041)	0.0473	0.0247	0.0226 (0.0123)	0.0629 (-1.8770), dS=dN
EGF1	0.0305 (0.0102)	0.1056	0.0131	0.0925 (0.0469)	**0.0424 (-2.052), dS>dN**
EGF2	0.0575 (0.0139)	0.0582	0.0616	-0.0034 (0.0397)	0.9327 (0.0846), dS=dN
EGF3	0.0193 (0.0079)	0	0.0251	-0.0251 (0.0101)	**0.0151 (2.4646), dS<dN**
EGF4	0.0203 (0.0087)	0.0409	0.0168	0.0241 (0.0231)	0.3025 (-1.0356), dS=dN
GPI anchor	0.0156 (0.0087)	0.0421	0.0076	0.0346 (0.0367)	0.3363 (-0.9654), dS=dN
***P. inui* (N=8)**					
Gene CDS	0.0133 (0.0026)	0.0088	0.0148	-0.0061 (0.0056)	0.2711 (1.1056), dS=dN
EGF1	0.0201 (0.0074)	0.0307	0.0180	0.0127 (0.0257)	0.6183 (-0.4995), dS=dN
EGF2	0.0177 (0.0066)	0.0087	0.0206	-0.0119 (0.0131)	0.3404 (0.9571), dS=dN
EGF3	0.0104 (0.0053)	0.0018	0.0129	-0.0111 (0.0067)	0.0752 (1.7951), dS=dN
EGF4	0.0020 (0.0020)	0	0.0025	-0.0025 (0.0025)	0.3268 (0.9845), dS=dN
GPI anchor	0.0195 (0.0093)	0.0132	0.0237	-0.0105 (0.0208)	0.5976 (0.5293), dS=dN
***P. knowlesi* (N=4)**					
Gene CDS	0.0038 (0.0015)	0.0071	0.0029	0.0042 (0.0051)	0.4180 (-0.8126), dS=dN
EGF1	0	0	0	0	1 (0), dS=dN
EGF2	0	0	0	0	1 (0), dS=dN
EGF3	0	0	0	0	1 (0), dS=dN
EGF4	0.0041 (0.0041)	0	0.0050	-0.0050 (0.0048)	0.3318 (0.9744), dS=dN
GPI anchor	0.0067 (0.0067)	0	0.0096	-0.0096 (0.0104)	0.3165 (1.0058), dS=dN

[a] (SE) Standard error
