# Supplementary material for: Evolution of the Transmission-Blocking Vaccine Candidates Pvs28 and Pvs25 in Plasmodium vivax: Geographic Differentiation and Evidence of Positive Selection
Source: PLoS Negl Trop Dis. 2016 Jun 27;10(6):e0004786. doi: 10.1371/journal.pntd.0004786 (PMC4922550; doi:10.1371/journal.pntd.0004786)
Supplement: S5 Table — (PDF) [file pntd.0004786.s005.pdf]

S5 Table. McDonald & Kreitman test for the *pvs28* and *pvs25* genes.

Gene	Species	Neutrality Index (p-values)	Fixed Syn	Fixed Non-Syn	Polymorphic (Syn)	Polymorphic (Non-Syn)
**P28**	*P. vivax* vs. *P. cynomolgi*	0.707 (0.5243)	10	41	20	58
	P. vivax vs. *P. inui*	2.213 (0.05157)	19	22	32	82
	P. vivax vs. *P. knowlesi*	1.864 (0.1376)	35	70	11	41
	P. cynomolgi vs. *P. inui*	0.745 (0.5540)	11	42	26	74
**P25**	***P. vivax* vs. *P. cynomolgi***	**2.738 (0.0125)***	**23**	**21**	**26**	**65**
	P. vivax* vs. *P. inui	**2.700 (0.0144)***	**21**	**28**	**15**	**54**
	P. vivax vs. *P. knowlesi*	1.672 (0.1940)	31	49	14	37
	P. cynomolgi vs. *P. inui*	1.472 (0.3940)	14	24	21	53
