# Supplementary material for: Evolution of the Transmission-Blocking Vaccine Candidates Pvs28 and Pvs25 in Plasmodium vivax: Geographic Differentiation and Evidence of Positive Selection
Source: PLoS Negl Trop Dis. 2016 Jun 27;10(6):e0004786. doi: 10.1371/journal.pntd.0004786 (PMC4922550; doi:10.1371/journal.pntd.0004786)
Supplement: S6 Table — (PDF) [file pntd.0004786.s006.pdf]

S6 Table. Likelihood ratio test statistics for Adaptive BS-REL and BUSTED analysis of *p28* and *p25* gene (18 and 17 species respectively).

Gene	Test: Adaptive BS-REL summary				
p28	**Evidence of episodic diversifying selection:** NO (LRT p ≤ 0.05, corrected for multiple testing.)				
	**ω rate classes**	**# of Branches**	**% of branches**	**% of tree length**	**# under selection**
	1	17	52%	0.11%	0
	2	16	48%	100%	0
	**Model**	**log L**	**# par.**	**AICc**	
	Branch-wise ω variation (MG94)	-6778.69	80	13720.89	
	Branch-site ω variation	-6665.79	128	13596.62	
p25	**Evidence† of episodic diversifying selection:** NO (LRT p ≤ 0.05, corrected for multiple testing.)				
	**ω rate classes**	**# of Branches**	**% of branches**	**% of tree length**	**# under selection**
	1	21	68%	0.43%	0
	2	10	32%	100%	0
	**Model**	**log L**	**# par.**	**AICc**	
	Branch-wise ω variation (MG94)	-5525.47	76	11206.08	
	Branch-site ω variation	-5457.98	106	11134.12	

Gene	Test: BUSTED (*P. falciparum* branch)					
p28	**Evidence of episodic diversifying selection:** SI (with LRT p-value =0.002)					
	**Model**	**log L**	**AICc**	**ω1**	**ω2**	**ω3**
	Unconstrained Model	-6716.34	13548.45	0.00 (87%)	0.00 (8.2%)	76.8 (4.8%)
	(background branches)			0.00 (40%)	0.280 (43%)	5.94 (16%)
	Constrained Model	-6722.68	13559.08	0.00 (6.6%)	0.00 (37%)	1.00 (57%)
p25	**Evidence of episodic diversifying selection:** NO (with LRT p-value =0.295)					
	**Model**	**log L**	**AICc**	**ω1**	**ω2**	**ω3**
	Unconstrained Model	-5503.91	11119.47	0.333 (71%)	0.731 (26%)	14.0 (2.4%)
	(background branches)			0.00 (60%)	0.833 (35%)	6.02 (4.7%)
	Constrained Model	-5505.13	11119.86	0.00 (43%)	0.00 (4.0%)	1.00 (53%)
