# Supplementary material for: Evolution of the Transmission-Blocking Vaccine Candidates Pvs28 and Pvs25 in Plasmodium vivax: Geographic Differentiation and Evidence of Positive Selection
Source: PLoS Negl Trop Dis. 2016 Jun 27;10(6):e0004786. doi: 10.1371/journal.pntd.0004786 (PMC4922550; doi:10.1371/journal.pntd.0004786)
Supplement: S7 Table — (PDF) [file pntd.0004786.s007.pdf]

S7 Table. Worldwide short tandem repeats in the *pvs28* gene and NHPPs orthologous genes.

Plasmodium spp.	Strain	[tandem repeat frequency]$_{\#}$
P. vivax	Sal-I	[GSGGE]$_3$-[GSGGD]$_1$
	Cosmopolitan[a]	[GSGGE]$_{2-6}$-[GSGGD]$_1$
	New Guinea	[ESGGE]$_1$-[GSGGE]$_4$-[GSGGD]$_1$
P. cynomolgi	B-007100	[GSGGQ]$_1$-[GSGGA]$_1$
	Mulligan	[GSGGQ]$_1$-[GSGRA]$_1$
	PT1	[GSGGQ]$_1$-[GSGGA]$_1$
	B(X-20)	[GSGGQ]$_1$-[GSGGA]$_1$
	RO	[GSGGE]$_1$-[GSGGQ]$_1$-[GSGGA]$_1$
	Berok	[GSGGE]$_{13}$-[GSGGA]$_1$
	PT2	[GSGGE]$_1$-[GSGGQ]$_1$-[GSGGA]$_1$
	B-062530	[GSGGQ]$_1$-[GSGGE]$_{2-}$-[GSGGQ]$_2$-[GSGGA]$_1$
	B-062510	[GSGGQ]$_1$-[GSGGG]$_1$-[GSGGQ]$_5$ -[GSGGA]$_1$
	B-007100	[GSGGQ]$_1$-[GSGGA]$_1$
	Ceylonensis	[GSGGE]$_1$-[GSGGQ]$_1$-[GSGGA]$_1$
	Smithsonian	[GSGGE]$_1$-[GSGGQ]$_1$-[GSGGA]$_1$
	Gombak	[GSGGV]$_1$-[GSGGE]$_1$-[GSGVE]$_{1-}$[GSGGA]$_1$
P. fieldi	N3	[EGSGE]$_1$-[GSGGE]$_3$
P. simiovale	-	[EGSGE]$_1$-[GSGGE]$_1$-[GNGGA]$_2$- [GNGGG]$_1$
P. inui	Celebes I	[ESGGE]$_1$-[GSGGE]$_1$-[GSGGA]$_1$
	Taiwan I	[ESGGE]$_1$-[GSGGE]$_4$-[GSGGA]$_1$
	Taiwan II	[ESGGE]$_1$-[GSGGE]$_4$-[GSGGA]$_1$
	N34	[ESGGE]$_1$-[GSGGE]$_2$-[GSGGA]$_1$
	Leucosphyrus	[ESGGE]$_1$-[GSGGE]$_2$-[GSGGA]$_1$
	Leaf Monkey II	[ESGGE]$_1$-[GSGGE]$_3$-[GSGGA]$_1$
	OS	[GSGGQ]$_1$-[GSGGE]$_2$-[GSGGA]$_1$
	Phillipine	[ESGGE]$_1$-[GSGGE]$_2$-[GSGGA]$_1$
	Perak	[ESGGE]$_1$-[GSGGE]$_2$-[GSGGA]$_1$
	Perlis	[ESGGE]$_1$-[GSGGE]$_1$-[GSGGA]$_1$
	Celebes II	[ESGGE]$_1$-[GSGGE]$_3$-[GSGGA]$_1$
P. hylobati	-	[ESGGE]$_1$-[GSGGE]$_6$-[GSGGA]$_1$
P. coatneyi	-	[GSGGA]$_1$-[GSGGE]$_1$-[GSGGD]$_1$
P. knowlesi	H	[GSGGQ]$_1$-[GSGGE]$_1$-[GNGGG]$_1$-[GSGGS]$_1$
	Hackeri	[GSGGQ]$_1$-[GSGGE]$_1$-[GNGGE]$_1$-[GSGGS]$_1$
P. gonderi	-	[GSGGG]$_1$-[GNGGE]$_1$-[GSGGE]$_1$-[SSGGE]$_1$
P. yoeli	-	[GTGS]$_1$-[GTGT]$_1$-[GSGT]$_1$-[GTGS]$_1$-[GTGT]$_1$
P. berghei	-	[GTGS]$_1$
P. gallinaceaum	-	[GSGSG]$_1$

Cosmopolitan[a]: field isolates from Bangladesh, China, Colombia, El Salvador, Honduras, India, Indonesia, Korea, Malaysia, Mauritania, Mexico, Nicaragua, Panama, Sumatra, Thailand, Venezuela and Vietnam. In the case of *P.cynomolgi* short tandem repeats for the paralogous genes were included.
