# Supplementary material for: Evolution of the Transmission-Blocking Vaccine Candidates Pvs28 and Pvs25 in Plasmodium vivax: Geographic Differentiation and Evidence of Positive Selection
Source: PLoS Negl Trop Dis. 2016 Jun 27;10(6):e0004786. doi: 10.1371/journal.pntd.0004786 (PMC4922550; doi:10.1371/journal.pntd.0004786)
Supplement: S8 Table — (PDF) [file pntd.0004786.s008.pdf]

S8 Table. Polymorphism in the repetitive motif of the Pvs28 protein and closely NHPPs orthologous genes.

**Species**	**Repeats**	**∏ (SE)**	**Ds**	**Dn**	**Ds-Dn (SD.)**	***p* (Z-stat)**
P. vivax (N=284)	[(**G**/E/S)**S**(**G**/R/D)**GE**]$_{2-6}$	0.0800 (0.0411)	0.5730	0.0016	0.5714 (0.2311)	**0.0172 (-2.4159), dS>dN**
P. cynomolgi (N=7) Gene PCYB_007100	[**GSGG(E**/Q**)**]$_{1-13}$	0.1111 (0.0464)	0.5267	0.0402	0.4886 (0.2317)	**0.01980 (-2.3616), dS>dN**
P. cynomolgi (N=4) Gene PCYB_062530	[**GSG(G**/V**)(E**/Q/V**)**]$_{2-5}$	0.1444 (0.0613)	0.5549	0.0797	0.4756 (0.3190)	0.1031 (-1.6423), dS=dN
P. inui (N=12)	[(**G**/E)**SGG(E**/Q**)**]$_{2-5}$	0.1162 (0.0498)	0.4505	0.0566	0.3972 (0.2253)	**0.0155 (-2.4556), dS>dN**
P. knowlesi (N=5)	[**G(S**/N**)GG(E**/Q/G**)**]$_{3}$	0.1924 (0.0642)	0.6009	0.1083	0.5394 (0.2885)	**0.0122 (-2.5435), dS>dN**
