# Supplementary figures and images for: Evolution of the Transmission-Blocking Vaccine Candidates Pvs28 and Pvs25 in Plasmodium vivax: Geographic Differentiation and Evidence of Positive Selection
Source: PLoS Negl Trop Dis. 2016 Jun 27;10(6):e0004786. doi: 10.1371/journal.pntd.0004786 (PMC4922550; doi:10.1371/journal.pntd.0004786)

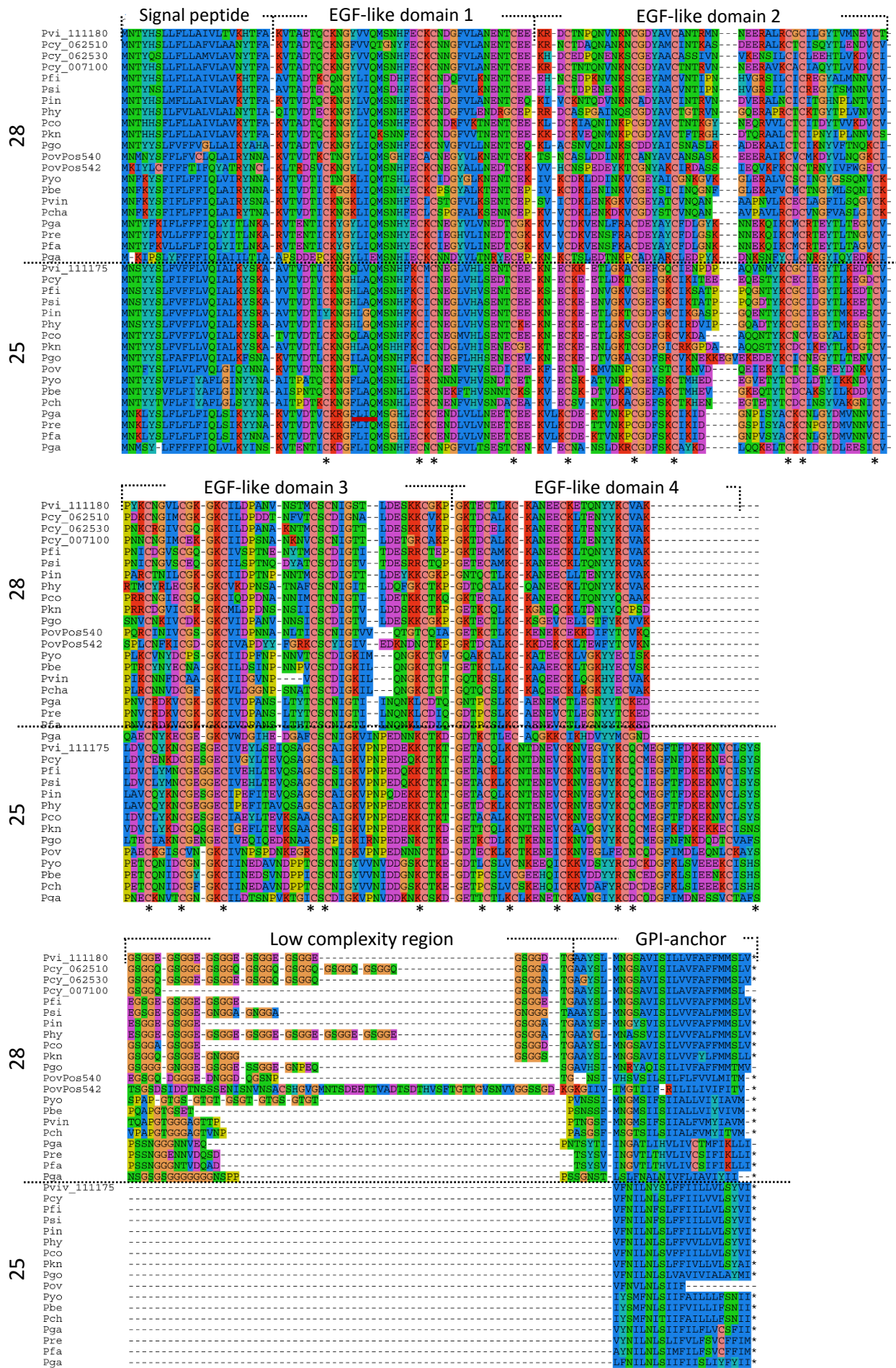

Supplement: S1 Fig — All genes shared a similar gene structure consisting of a signal sequence of 23 amino acids at the N-terminus followed by four cysteine-rich EGF-like domains and a short GPI anchor region at the C-terminus. Cysteines are conserved among Plasmodium spp. The species names are abbreviated as follow: Pv, P. vivax; Pcy, P. cynomolgi; Pfi, P. fieldi; Psi, P. simiovale; Pin, P. inui; Phy, P. hylobati; Pco, P. coatneyi; Pk, P. knowlesi; Pgo, P. gonderi; Pov, P. ovale; Pyo, P. yoelii; Pbe, P. berghei; P. vin, P. vinckei; Pcha, P. chabaudi; Pga, P. gallinaceum; Pre, P. reichenowi; Pf, P. falciparum, and Pga, P. gaboni. (PDF) [file pntd.0004786.s009.pdf]

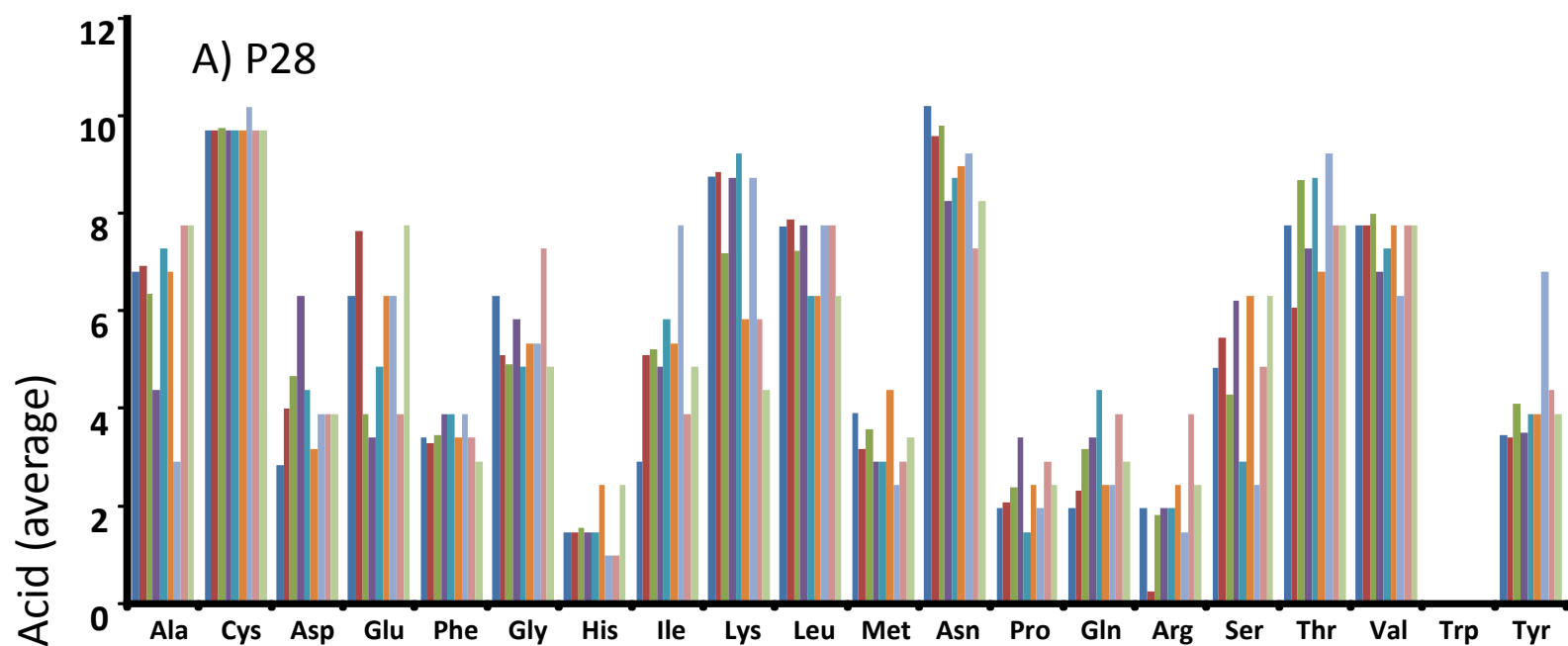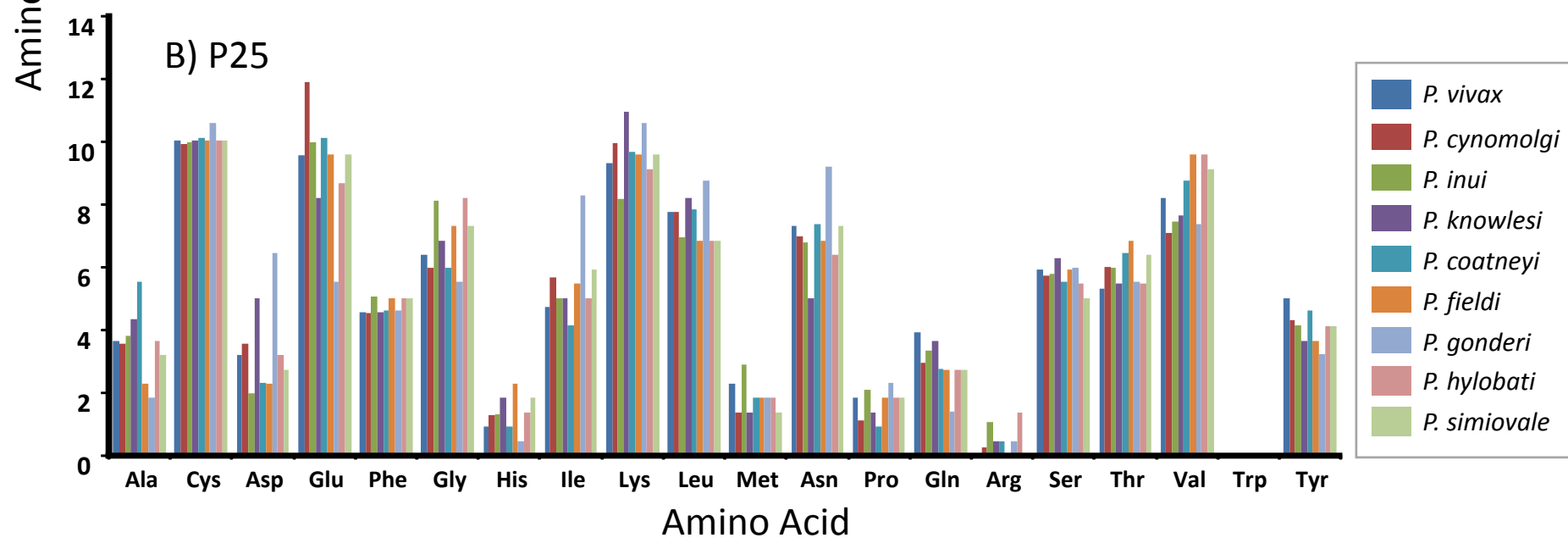

Supplement: S2 Fig — Amino acid composition of the P28 (A) and P25 (B) proteins for P. vivax and closely NHP malarias. (PDF) [file pntd.0004786.s010.pdf]

### A) P28

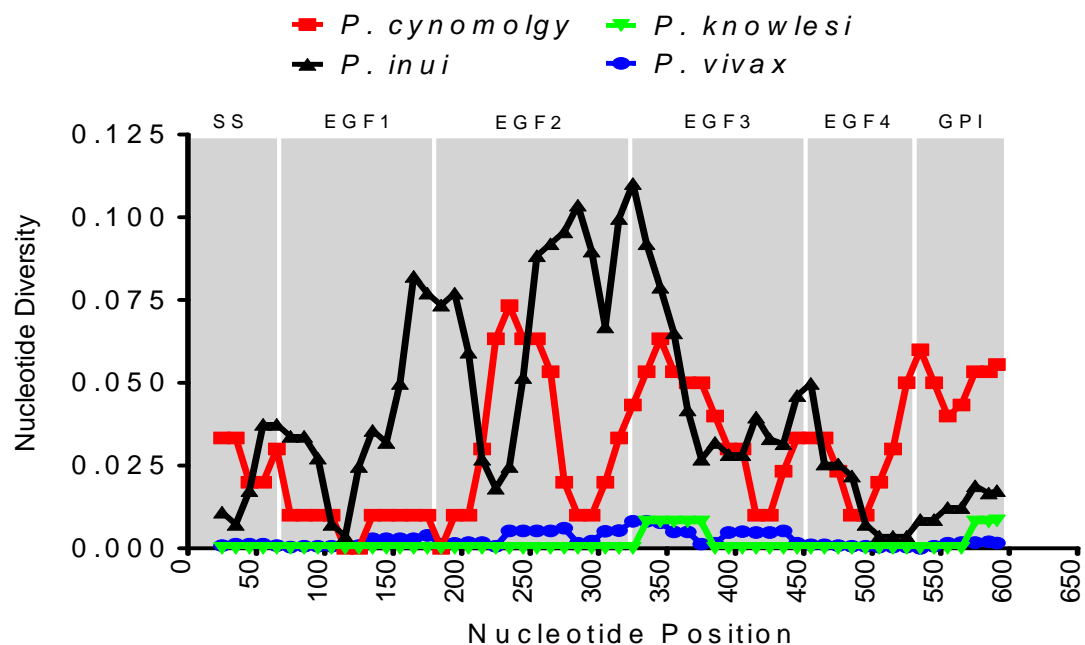

### B) P28 (*P. cynomolgy*)

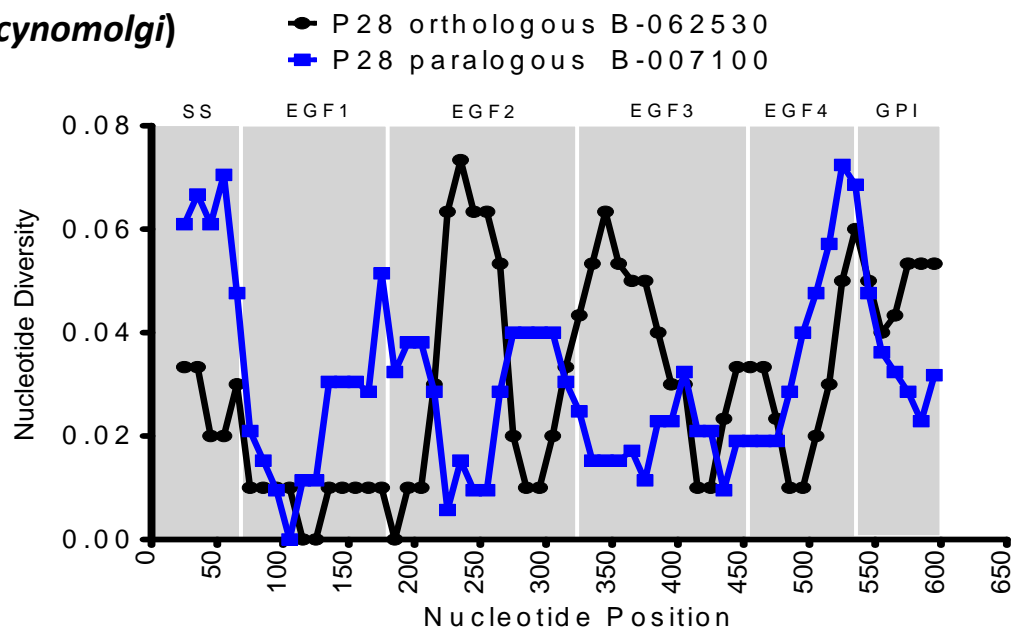

### C) P25

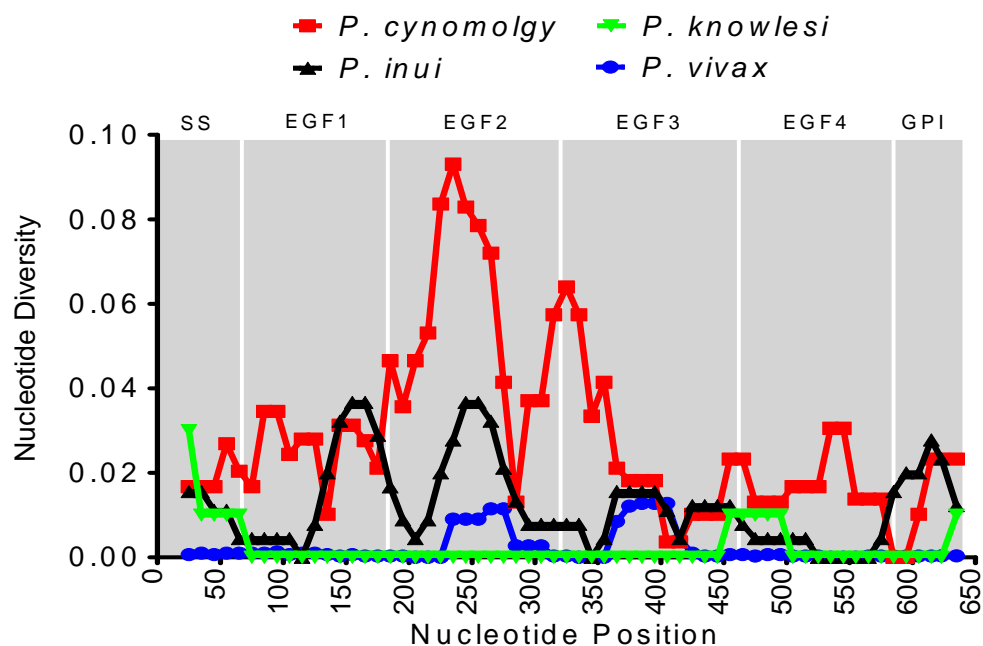

Supplement: S3 Fig — Sliding window analysis of the nucleotide diversity (π) of the p28 gene (A), P. cynomolgi p28 paralogous genes (B), and the p25 gene (C). The genetic diversity was estimated by calculating (π) on a window of 50 base pairs moving it in steps of 10 sites. LCR were not included. (PDF) [file pntd.0004786.s011.pdf]

A) p28

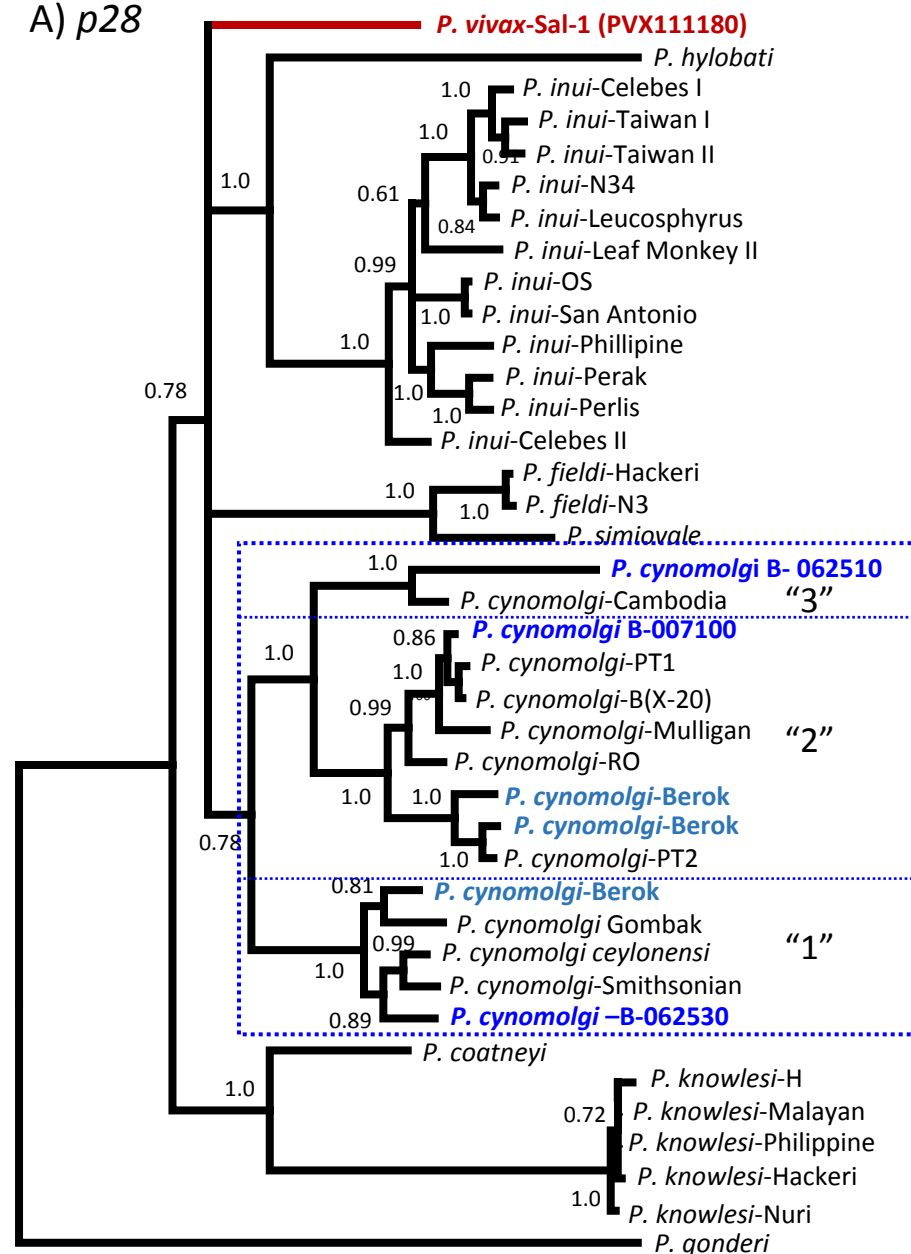

B) p25

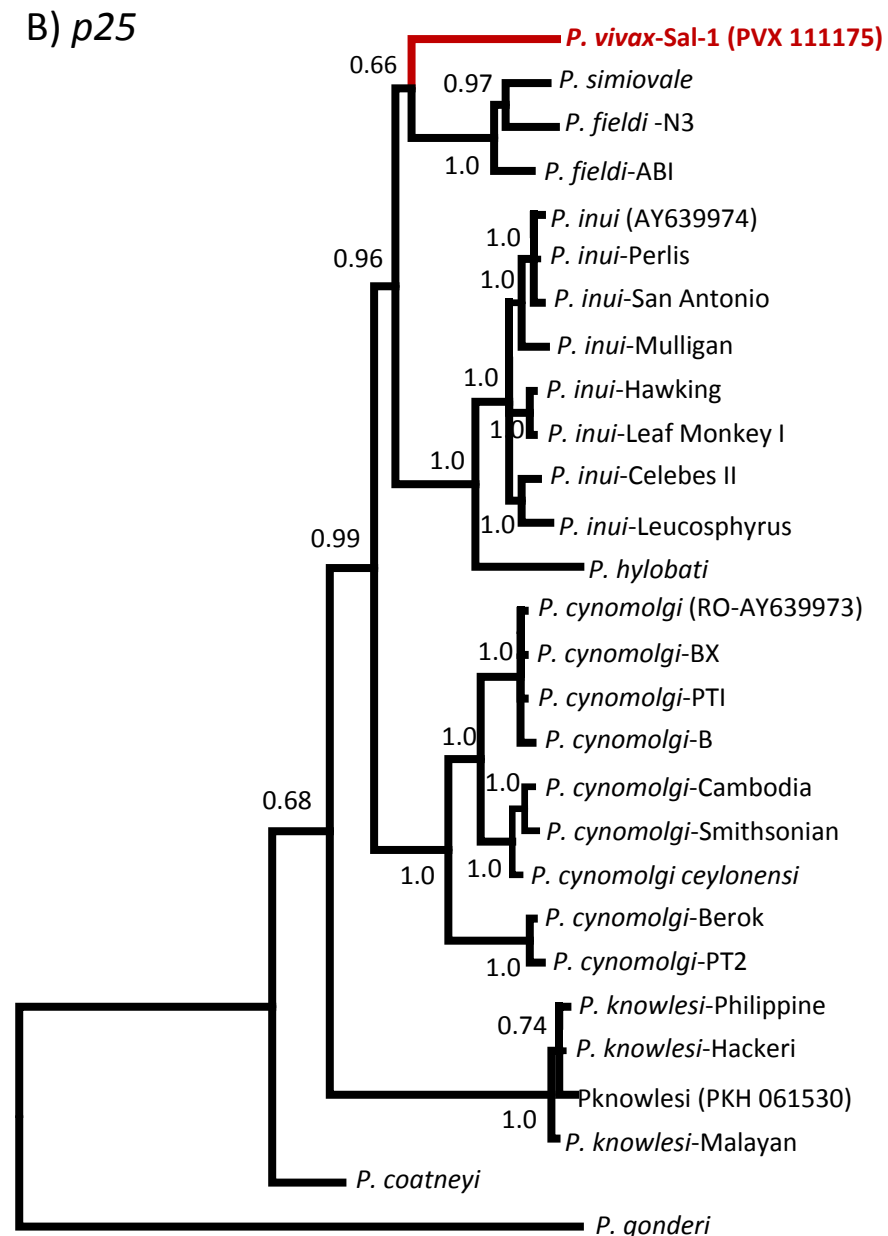

Supplement: S4 Fig — Bayesian phylogenetic hypothesis constructed using nucleotide sequences of the genes encoding the P28 (A) and P25 (B) antigens from Plasmodium spp. and strains amplified in this study. The values above branches are posterior probabilities (see Methods section). For both phylogenies (p28 and p25), two independent chains were sampled every 200 generations in runs lasting 6 × 10^6^ Markov Chain Monte Carlo steps. In the case of P28 (A), the clade of P. cynomolgi consists of three subgroups (1–3). “1” refers to the lineage “PCYB-062530”, which includes strains of its orthologous gene (Ceylonensi, Smithsonian, Gombak). The “2” and “3” subgroups refer to the lineages “PCYB-007100” (Mulligan, PT-1, BX-20, RO) and “PCYB-062510” (Cambodia) respectively, which contain strains corresponding to its paralogous genes. (PDF) [file pntd.0004786.s012.pdf]
